# Graphene binding on black phosphorus enables high on/off ratios and mobility

**DOI:** 10.1093/nsr/nwad279

**Published:** 2023-11-03

**Authors:** Fanrong Lin, Zhonghan Cao, Feiping Xiao, Jiawei Liu, Jiabin Qiao, Minmin Xue, Zhili Hu, Ying Liu, Huan Lu, Zhuhua Zhang, Jens Martin, Qingjun Tong, Wanlin Guo, Yanpeng Liu

**Affiliations:** Key Laboratory for Intelligent Nano Materials and Devices of Ministry of Education, State Key Laboratory of Mechanics and Control of Mechanical Structures, and Institute for Frontier Science, Nanjing University of Aeronautics and Astronautics, Nanjing210016, China; Centre for Advanced 2D Materials, National University of Singapore, Singapore 117546, Singapore; School of Physics and Electronics, Hunan University, Changsha410082, China; Centre for Advanced 2D Materials, National University of Singapore, Singapore 117546, Singapore; Centre for Quantum Physics, Key Laboratory of Advanced Optoelectronic Quantum Architecture and Measurement, School of Physics, Beijing Institute of Technology, Beijing100081, China; Key Laboratory for Intelligent Nano Materials and Devices of Ministry of Education, State Key Laboratory of Mechanics and Control of Mechanical Structures, and Institute for Frontier Science, Nanjing University of Aeronautics and Astronautics, Nanjing210016, China; Key Laboratory for Intelligent Nano Materials and Devices of Ministry of Education, State Key Laboratory of Mechanics and Control of Mechanical Structures, and Institute for Frontier Science, Nanjing University of Aeronautics and Astronautics, Nanjing210016, China; Key Laboratory for Intelligent Nano Materials and Devices of Ministry of Education, State Key Laboratory of Mechanics and Control of Mechanical Structures, and Institute for Frontier Science, Nanjing University of Aeronautics and Astronautics, Nanjing210016, China; Key Laboratory for Intelligent Nano Materials and Devices of Ministry of Education, State Key Laboratory of Mechanics and Control of Mechanical Structures, and Institute for Frontier Science, Nanjing University of Aeronautics and Astronautics, Nanjing210016, China; Key Laboratory for Intelligent Nano Materials and Devices of Ministry of Education, State Key Laboratory of Mechanics and Control of Mechanical Structures, and Institute for Frontier Science, Nanjing University of Aeronautics and Astronautics, Nanjing210016, China; Leibniz Institute für Kristallzüchtung, Berlin12489, Germany; School of Physics and Electronics, Hunan University, Changsha410082, China; Key Laboratory for Intelligent Nano Materials and Devices of Ministry of Education, State Key Laboratory of Mechanics and Control of Mechanical Structures, and Institute for Frontier Science, Nanjing University of Aeronautics and Astronautics, Nanjing210016, China; Key Laboratory for Intelligent Nano Materials and Devices of Ministry of Education, State Key Laboratory of Mechanics and Control of Mechanical Structures, and Institute for Frontier Science, Nanjing University of Aeronautics and Astronautics, Nanjing210016, China

**Keywords:** graphene, on/off ratio, mobility, black phosphorus, reflective interface

## Abstract

Graphene is one of the most promising candidates for integrated circuits due to its robustness against short-channel effects, inherent high carrier mobility and desired gapless nature for Ohmic contact, but it is difficult to achieve satisfactory on/off ratios even at the expense of its carrier mobility, limiting its device applications. Here, we present a strategy to realize high back-gate switching ratios in a graphene monolayer with well-maintained high mobility by forming a vertical heterostructure with a black phosphorus multi-layer. By local current annealing, strain is introduced within an established area of the graphene, which forms a reflective interface with the rest of the strain-free area and thus generates a robust off-state via local current depletion. Applying a positive back-gate voltage to the heterostructure can keep the black phosphorus insulating, while a negative back-gate voltage changes the black phosphorus to be conductive because of hole accumulation. Then, a parallel channel is activated within the strain-free graphene area by edge-contacted electrodes, thereby largely inheriting the intrinsic carrier mobility of graphene in the on-state. As a result, the device can provide an on/off voltage ratio of >10^3^ as well as a mobility of ∼8000 cm^2^ V^−1^ s^−1^ at room temperature, meeting the low-power criterion suggested by the International Roadmap for Devices and Systems.

## Teaser

In order to overcome the trade-off between mobility and on/off ratio in monolayer graphene, selectively relaxing graphene stacked on black phosphorus was rationally developed toward future electronic applications.

## INTRODUCTION

Field effect transistors (FETs) are the core elements of current integrated circuits [1–3]. Conventional silicon-based FETs are facing critical issues when scaled down to nanometers, such as self-heating [4], charge leakages [5] and short-channel effects [3,6]. To address these issues, it is of paramount importance to achieve a high on/off ratio that permits effective logical operation at the nanometer size limit and meanwhile hold high carrier mobility that enables low power consumption [7]. To this end, extensive efforts have been devoted to developing new nanomaterials and designing innovative device architectures [8,9].

Graphene—a single layer of carbon atoms in a honeycomb lattice—exhibits outstanding carrier mobility (>10^4^ cm^2^ V^−1^ s^−1^) [10–12], negligible contact resistance [13,14] and high compatibility with conventional manufacturing processes that mark it as a promising candidate for downscaling logic devices. Yet, the on/off ratio of graphene is commonly <30 due to its gapless nature and this limits its application in logic devices [3,15–22]. Several strategies have been reported to increase the on/off ratios by opening a bandgap in the graphene but often at the expense of significantly reduced carrier mobility [23]. For example, patterning graphene into nanoribbons [24] and functionalizing graphene [25–30] can open a sizable bandgap yielding a high on/off ratio of ≤70 but the carrier mobility drops to <880 cm^2^ V^−1^ s^−1^ [24]. In fact, the challenge in achieving a good trade-off between the on/off ratio and carrier mobility has been encountered in silicon and 2D semiconductors as well [31]. Therefore, it is imperative to find a new approach (especially in engineering device architecture) that can provide high on/off ratios while, to a great extent, maintaining the intrinsic carrier mobility.

Here, we report a new device architecture consisting of a graphene monolayer bound on a black phosphorus (BP) multi-layer, which can offer an on/off ratio of >10^3^ and a mobility close to the intrinsic limit of graphene. In particular, we introduce hetero-strain in graphene by local current annealing that results in a reflective interface between the strained and unstrained graphene areas and thus a robust off-state against external magnetic fields and elevated temperature. Then, an applied bias voltage can open a conductive channel in the BP that in turn switches an on-state in the graphene through the electrodes edge-contacting all the atomic layers. Such a device architecture is, in principle, applicable to any high-mobility 2D materials bound on a range of semiconducting multilayers and may indicate a new paradigm for fabricating high-performance electronic devices.

## GENERATING A REFLECTIVE INTERFACE IN A DUAL-GATED GRAPHENE TRANSISTOR

**Figure 1.**
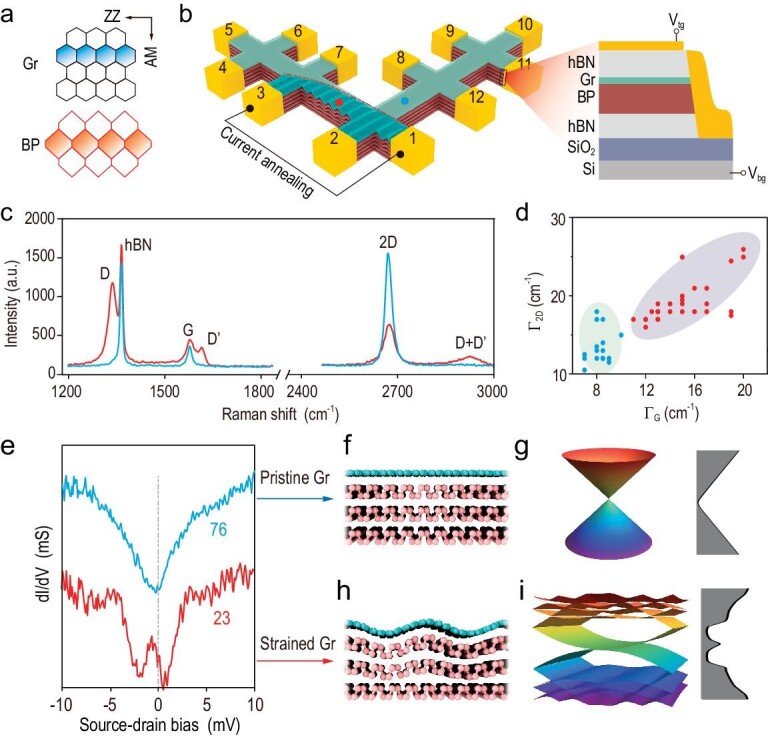
Dual-gated graphene transistor. (a) Atomic structure of Gr (top panel) and BP (bottom panel). (b) Schematic of graphene transistor. The flat and rippled regions represent unannealed and annealed Gr, respectively. The right panel sketches the side view of the transistor. (c) Raman spectra of unannealed (blue curve) and annealed (red curve) sample regions. For clarification, the peaks at 1365.7 cm^−1^ originate from the in-plane mode of the hBN crystal [51]. (d) The FWHMs of G and 2D peaks collected from the unannealed (blue dots) and annealed (red dots) regions. (e) Differential conductance vs applied DC-bias plots for unannealed (Electrodes 6 and 7) and annealed (Electrodes 2 and 3) sample regions at 1.5 K. Curves were vertically shifted for clarity. Schematic of (f) atomic structure of pristine Gr/BP stack and (g) simulated Gr band structure. (h) Proposed structure of annealed Gr/BP region and (i) corresponding band structure of Gr.

The device consists of a graphene monolayer stacked on BP (thickness ∼23 nm), which are overall sandwiched between two hexagonal boron nitride (hBN) sheets. The zigzag directions of Gr and BP crystals are well aligned (Fig. 1a), while the twist angle between the Gr and the hBN was set at ∼15° to diminish unfavorable interlayer interaction. Then, the hBN/Gr/BP/hBN stack was etched to form an L-shaped device (Fig. 1b) with two channels parallel and perpendicular to the zigzag direction of the BP, respectively. After that, the graphene-based transistor (right panel, Fig. 1b) was completed by depositing 12 electrodes (indexed as 1–12, see Methods) that edge-contacted all the atomic layers [13]. The dual gates were realized by using a gold top-gate and a silicon back-gate [32,33].

A key feature in the as-fabricated transistor is the in-plane reflective interface between the strained and unstrained graphene areas. The strained area between Electrodes 1 and 3 (Fig. 1b) was created via local current annealing (at 1 mA/μm) for 120 min at 300 K [34–36]. In our previous work [37], graphene on multi-layer BP after thermal annealing was found to undergo non-uniform lattice strain resulting from large lattice mismatch. Figure 1c shows two representative Raman spectra collected from unannealed and annealed sample regions. The full widths at half maximum (FWHM) of the G peak (at ∼1576.9 cm^−1^) and 2D peak (at ∼2677.9 cm^−1^) corresponding respectively to high-frequency *E*_2__g_ phonon and double-resonance modes broaden for annealed graphene. In accordance with statistical analysis of 38 spectra (*Γ*_G_ and *Γ*_2D_, Fig. 1d), the FWHMs of the G and 2D peaks of the annealed graphene increase to 15 ± 4 and 20 ± 4 cm^−1^ (red scatters) from 8 ± 2 and 14 ± 4 cm^−1^ (blue scatters, unannealed region), respectively, evidencing occurrences of local lattice strain after local annealing. Furthermore, the robust D (1340.3 cm^−1^), D' (1615.4 cm^−1^) and D + D' (2932.7 cm^−1^) peaks, which are correlated with the breathing modes of the sp^2^ rings, are clearly observed in the annealed region [38–40]. This enhanced backscattering phenomenon of photo-excited electrons/holes reveals feasible moiré potentials or nano-rippled structures after thermal treatment. In the meantime, charge localization may also be more likely to occur within annealed graphene and produce a reflective interface at the in-plane strain junction (see Supplementary Fig. S3) [41,42].

To unravel the electronic variations induced by local annealing, the differential conductance d*I*_ij_/d*V* (*i* and *j* represent selected electrodes, Fig. 1b) of a graphene-based transistor as a function of DC-bias (*V*_bias_) was conducted at 1.5 K. The d*I*_76_/d*V* curve (Fig. 1e) of unannealed graphene shows a ‘V’ shape with its minimum at *V*_bias_ = 0 V, consistently with the typical transport behavior of intrinsic graphene [21]. In contrast, the d*I*_23_/d*V* curve (annealed region) reveals a robust peak at |*V*_bias_| < 1.5 mV, presumably originating from the charge localization by annealing-induced strain and lattice distortion (see Supplementary Section S2.9). To gain more insights, we utilized a parametrizing strain as a pseudo-gauge field to calculate the electronic structure of strained (annealed) and pristine (unannealed) graphene (see Supplementary Section S1.4). The band dispersion of unannealed graphene on BP (Fig. 1f and g) remains linear but becomes nearly flat under strain (Fig. 1h and i). The theoretical reproduction of differential conductance nicely validates the creation of a reflective interface within the in-plane junction area by local annealing.

## UNCONVENTIONAL LONGITUDINAL AND TRANSVERSE TRANSPORT

**Figure 2.**
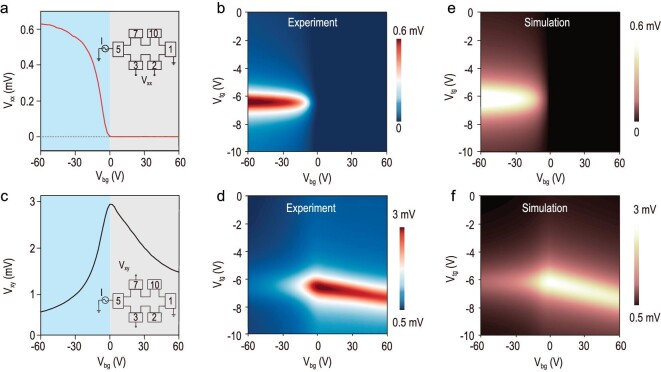
Unconventional voltage transition in graphene transistor at 120 K and *I*_bias_ = 100 nA. (a) Longitudinal voltage (*V*_xx_, Electrodes 3 and 2) as a function of *V*_bg_. The inset shows the corresponding configuration. (b) Experimental dual-gated map of *V*_xx_. (c) Transverse voltage (*V*_xy_, Electrodes 7 and 3) as a function of *V*_bg_. The inset sketches the configuration for *V*_xy_. (d) Experimental dual-gated map of *V*_xy_. Simulated dual-gated maps of (e) *V*_xx_ and (f) *V*_xy_, respectively.

We next explored the transport behavior of a graphene-based transistor in the Hall-bar configuration, inserted in Fig. 2a. After injecting current (100 nA) from Electrode 5 to Electrode 1, the longitudinal voltage (*V*_xx_, Electrodes 3 and 2) curve shows an asymmetric transition from approximate zero (*T* = 120 K, signal noise level < 0.02 μV limited by lock-in measurement) for a positive back-gate voltage (*V*_bg_ ≥ 0 V) to hundreds of microvolts for a negative one (*V*_bg_ < 0 V), in contrast to the typical V-shaped curve of intrinsic graphene reported in previous literature [12,13,21]. Furthermore, the voltage on/off ratio was calculated to be ≥3 × 10^4^ even by considering the noise as the off-state signal. Furthermore, the dual-gated mapping of *V*_xx_ (Fig. 2b) exhibits that *V*_xx_ always approaches zero in the region of *V*_bg_ ≥ 0 V. When *V*_bg_ is <0 V, the *V*_xx_ rises with the well-known Dirac features, suggesting the dominant role of graphene in total transport. Meanwhile, the carrier mobility extracted was ∼20 000 cm^2^ V^−1^ s^−1^ at *T* = 120 K (see Supplementary Section S2.5). Moreover, the voltage at the Dirac point scales up with decreasing *V*_bg_ because the *V*_bg_-activated conducting BP channel screens the *V*_bg_ and contributes to the total output voltage (detailed discussion in later section).

It is known that the transverse signal of ideal graphene is negligible in the absence of a magnetic field due to the weak spin–orbital coupling in graphene [43]. In contrast, the transverse voltage (*V*_xy_, Electrodes 7 and 3, inset in Fig. 2c) here is non-zero and reaches the maximum at *V*_bg_ = 0 V. In addition, the transverse voltage vs *V*_tg_ curves (Fig. 2d) show a typical Dirac feature at different *V*_bg_, indicating an inhomogeneous current flow within the graphene. The finite slope (d*V*_tg_/d*V*_bg_) of the Dirac point at *V*_bg_ ≥ 0 V suggests that the displacement field from the back-gate voltage partially penetrates the BP to modulate the carrier density of the graphene. While *V*_bg_ < 0 V, the d*V*_tg_/d*V*_bg_ turns into zero resulting from complete screening of *V*_bg_ by the BP layers.

To theoretically quantify the longitudinal and transverse responses, we developed a parallel-transport model by incorporating an in-plane junction and gate-switchable electrical connection. We first defined a parallel propagation coefficient that characterizes the transverse signal reduction by the increment of BP conductivities:


(1) \begin{eqnarray*}\eta \left( {{V}_{{\mathrm{bg}}}} \right) = {V}_{51,73}\left( {{V}_{{\mathrm{bg}}}} \right)/{V}_{51,73}\left( 0 \right),
\end{eqnarray*}

where subscripts 51 and 73 represent the injecting current from Electrodes 5 to 1 and capturing the voltage difference between Electrodes 7 and 3. *η* equals 1 for insulating BP (*V*_bg_ ≥ 0 V) and drops with increasing BP conductivity from hole accumulations (*V*_bg_ < 0 V). Considering the electrostatic screening effect, we derived the following function:


(2) \begin{eqnarray*}{V}_{xx} = \left( {1 - \eta } \right){V}_{51,710},
\end{eqnarray*}

which links the unstrained and strained (annealed) graphene through the conducting BP layer. Modified by the parallel propagation coefficient, therefore, the *V*_xx_ at the on-state actually reflects the voltage variation in pristine graphene (*V*_51,710_). The independence between *η* and *V*_tg_ determines the on-state function *V*_xx_(*V*_tg_) following the same manner as *V*_51,710_(*V*_tg_). Additionally, the electronic structure of unannealed graphene remains uninterrupted and dominates the high carrier mobility of graphene devices. On this basis, the simulated evolutions of both *V*_xx_ and *V*_xy_ (Fig. 2e and f) with dual gates perfectly match with our experimental data (Fig. 2b and d). The gate-switchable conduct–insulate states of the BP and the electrical connection contribute to the concurrence of a high on/off ratio and mobility in the graphene monolayer.

## PROPOSED WORKING PRINCIPLES

**Figure 3.**
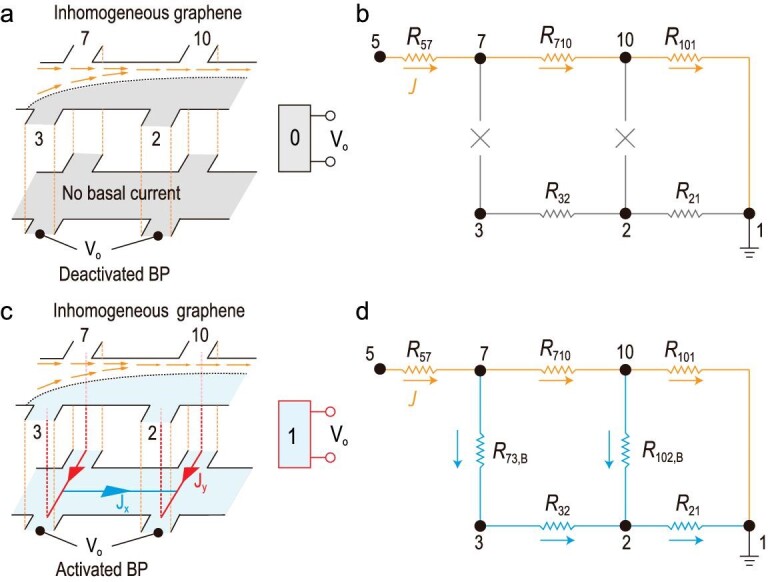
Operation mechanism of graphene-based transistor. (a) Inhomogeneous current flow within Gr channel. When BP is insulating, the voltage of annealed Gr is zero, representing Boolean ‘0’ state. (b) Effective resistor network for Boolean ‘0’ state. Current path is highlighted in orange. (c) Dual-channel propagations of graphene and BP bottom layer. Non-zero *V*_o_ represents Boolean ‘1’ state. Electrodes bridge graphene and BP bottom channels at each pin point denoted as red dashed lines. Red solid lines represent bridge currents at BP bottom channel. (d) Equivalent resistor network for Boolean ‘1’ state. *R*_73, B_ and *R*_102, B_ denote the resistance of the BP bottom channel. The activated current channel is highlighted in blue.

In accordance with the electrostatic screening effect, our device exhibits a surface propagation mode whereby the charges are electrically polarized at the top graphene/BP interface and bottom BP/hBN interface [33]. Graphene possesses better conductivity at the top interface and dominates the charge transport especially at low operation bias. For simplicity, we employed graphene to represent the top interface (see Supplementary Section S2.3). At the bottom interface, accumulating surface charges at the BP/hBN interface enable bottom BP layer conductivity [44,45]. When *V*_bg_ ≥ 0 V, the Fermi energy lies within the BP bandgap and the bottom BP channel is depleted (Fig. 3a). After injecting current flow from Electrode 5 to Electrode 1, electrons are restricted within the unannealed region (orange path, Fig. 3b) by the reflective interface, producing a zero voltage potential between the Electrodes 3 and 2 and grounded Electrode 1. Meanwhile, the transverse *V*_xy_ referring to the electrostatic potential at Electrode 7 is governed by the graphene resistance (*R*_71_) and becomes non-zero (Fig. 2c and d). This hypothesis was reconfirmed using inter-electrode Landauer–Büttiker transmissions that prohibit charge transmission across the reflective interface within an in-plane strain junction (see Supplementary Section S2.6). One superiority of the Gr/BP interface lies in the formation of an in-plane reflective interface via partially annealing to guide the charge flow and regulate the voltage.

When *V*_bg_ < 0 V, the bottom BP channel becomes conductive and propagates in parallel with the graphene. The thickness of the conducting BP bottom channel is estimated to be ∼3 nm due to the well-known screening effect [46]. More importantly, the edge-contact electrodes connect Electrode 3 (annealed region) and Electrode 7 (unannealed region) via the bottom BP channel. Note that the charge tunneling probability between the graphene and the bottom BP channel exponentially decays with increasing BP thickness and becomes ignorable for a thick tunneling barrier (>10 nm) in our devices. Consequently, a Hall-like transverse current (*J*_y_) flow in the bottom BP channel (red solid lines in Fig. 3c) balances the electrostatic potential difference over the reflective interface. Non-zero *I*_32_ as well as *V*_32_ is then developed, as demonstrated by the equivalent circuit illustrated in Fig. 3d. Guided by this, we are able to switch the bottom BP channel from the off-state into highly mobile on-state by using the back-gate toward high on/off ratios in graphene.

## ROBUSTNESS AGAINST MAGNETIC FIELD AND TEMPERATURE

**Figure 4.**
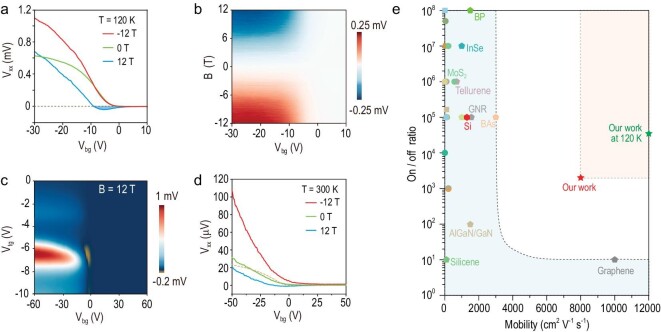
Device performance at different magnetic fields and temperature. (a) *V*_xx_ as a function of *V*_bg_ for *B* = −12 T (red), 0 T (green) and 12 T (blue) at *T* = 120 K and *V*_tg_ = −6.2 V. The negative *V*_xx_ that emerged at −8 V < *V*_bg_ < 0 V is due to a dedicated interplay between the magnetic-field-induced Hall signal and the strain-junction-induced Hall-like one in the BP bottom channel. (b) Extracted *V*_as__–__xx_ as functions of *B* and *V*_bg_. (c) Dual-gated map of *V*_xx_ at *T* = 120 K and *B* = 12 T. Negative *V*_xx_ is highlighted in orange. (d) Magnetic-field-dependent *V*_xx_–*V*_bg_ evolution at *T* = 300 K and *V*_tg_ = 0 V. The curves in red, green and blue correspond to the data collected at *B* = −12, 0 and 12 T, respectively. For comparison, the data collected at *T* = 120 K and *V*_tg_ = 0 V (dashed orange curve) is also presented. (e) Room-temperature on/off ratio and carrier mobility of FETs based on various semiconductors including metal oxides, transition metal dichalcogenides and puckered semiconductors (Supplementary Table S1).

To simulate real-world working conditions, the graphene-based transistor was exposed to magnetic fields (*B* = ±12 T) and the *V*_xx_–*V*_bg_ curves (Fig. 4a) at *T* = 120 K reveal two notable features. First, the transistor remains in the off-state at *V*_bg_ ≥ 0 V even when being exposed to perpendicular magnetic fields. This indicates that the in-plane strain junction is robust and deflects all the charges even under cyclotron motions and Hall fields. Second, the magnitude of the on-state responses at ±12 T evolves differently for *V*_bg_ < 0 V and becomes negative at +12 T in the range of −8 V < *V*_bg_ < 0 V. To gain a better understanding of the asymmetric magneto-conductance, we mapped the magneto-asymmetric component *V*_as__–__xx_ as functions of the magnetic field and *V*_bg_ (Fig. 4b), where *V*_as__–__xx_ was extracted according to ${V}_{{\mathrm{as}} - {\mathrm{xx}}} = ( {{V}_{{\mathrm{xx}}}( B ) - {V}_{{\mathrm{xx}}}( { - B} )/2} )$. For the on-state, *V*_as__–__xx_ exhibits a sign reversal when switching magnetic fields from negative to positive, which is identical to the Hall effect of a bridge current. Therefore, the overall output voltage is the superposition of the voltage component from *J*_x_ and the Hall-like signal from *J*_y_, revalidating our assumption of the parallel propagation model (Fig. 3c). Figure 4c shows the 2D mapping of *V*_xx_ as functions of dual gates at *B* = 12 T and the ideal off-state (at *V*_bg_ ≥ 0 V) demonstrates the robustness of our transistor against magnetic disturbances.

In addition to the magnetic-field considerations presented above, the off-state of the graphene-based transistor (Fig. 4d) remained near zero at room temperature, resulting in an impressive on/off ratio of up to ∼2 × 10^3^, even when the noise voltage of ∼0.02 μV (limited by lock-in instruments, *I*_bias_ = 100 nA) was taken into account when estimating the off-state signal. Additionally, the room-temperature electron mobility of the graphene-based transistor was determined to be ∼8000 cm^2^ V^−1^ s^−1^. Here, the bridge current relies heavily on the Schottky barrier between the BP and metal contacts. The bandgap of the BP was found to increase monotonically with elevated temperature [47], which enlarged the Schottky barrier that constrains thermally excited carriers, thus facilitating robust voltage switching at room temperature.

Figure 4e presents a comparison of the on/off ratio and mobility of our graphene-based transistor with those of typical semiconducting material devices reported in previous literature. Although transition metal dichalcogenides typically exhibit on/off ratios of >10^5^, their mobility (200–410 cm^2^ V^−1^ s^−1^) is severely limited due to the strong phonon scattering [48] as compared with the predicted mobility values for silicon, which are higher for holes (∼650 cm^2^ V^−1^ s^−1^) and electrons (∼1400 cm^2^ V^−1^ s^−1^) [49]. Toward high on/off ratios, our graphene-based transistor employs a semiconducting BP channel and an in-plane strain junction, while a non-annealed graphene channel delivers exceptional mobility, surpassing the L-region of prior performance data. Simultaneously achieving a high on/off ratio and carrier mobility offers the potential for reducing power consumption in high-performance integrated circuits.

## CONCLUSIONS

In conclusion, we have developed a graphene-based transistor that boasts an excellent on/off ratio (>10^3^) and high carrier mobility (∼8000 cm^2^ V^−1^ s^−1^) at room temperature. It is generally believed that achieving a complete off-state and high on/off ratio is not feasible for high-mobility graphene. We show that local annealing of monolayer graphene, interfaced with substrates that are mismatched in terms of symmetry and crystal lattice, can result in a near-zero off-state. Notably, the resulting high on/off ratio is robust against magnetic fields and elevated temperatures, and is voltage-based, which can be conveniently converted into a current-based ratio, in accordance with Ohm's law for diverse application scenarios. Moreover, the off-states are constrained by the noise signal level determined by the lock-in measurement, indicating that an upsurge in current bias can lead to a higher on-state signal and a greater on/off ratio. While our research has only examined the combination of graphene and BP, it is expected that overlaying graphene on black arsenic, SnSe and their homologs will yield similar coexistence of a high on/off ratio and carrier mobility in a single graphene transistor at room temperature. This development will enable graphene transistors to be integrated into modern circuits with reduced power consumption.

## METHODS

### Fabrication of the graphene-based devices

The cleavage of layered material sheets onto SiO_2_/Si substrates was achieved utilizing blue ‘magic’ tape. An exfoliated BP or graphene flake with a straight edge and well-defined geometry was selectively utilized. Raman mapping was then adopted to distinguish the zigzag or armchair nature of the crystal edges. To avoid any degradation, both the exfoliation and the transfer processes involving the BP were executed within a glove box under an argon atmosphere, with the oxygen and water levels maintained at <0.1 ppm.

Following this, we adhered the hBN–graphene–BP heterostructure using a well-established dry-transfer technique [50]. We first prepared a hybrid film comprising 5 wt% of poly(bisphenol A carbonate) (PC) and 15 wt% of polypropylene carbonate (PPC). The PC layer served as an adhesive to pick up the 2D materials selectively while the PPC layer was liquefied to preclude the formation of interlayer bubbles. This hybrid film was then placed atop a polydimethylsiloxane (PDMS) stack for additional manipulation. Ultimately, we treated the PDMS surface with O_2_ plasma (Femto Plasma Etcher) under 50 sccm and 50 W for 1 min to enhance the surface adhesion with the PC/PPC film and prevent delamination during the transfer process.

The specific dry-transfer procedure is as follows: the top hBN layer was lifted at 70°C. Consecutively, the graphene and BP flakes were assembled together at 120°C. During the graphene/BP assembly, the zigzag edges of the two materials were aligned (0°) or misaligned (15°) using a well-established transfer platform with a rotatable plate. After that, the assembled heterostructure was finally released onto the SiO_2_/Si substrate at 180°C.

Subsequently, standard electron beam lithography was carried out to define the device geometry using a polymethyl methacrylate mask, followed by CHF_3_/O_2_ ion etching. Cr/Au (3/70 nm) was thermally deposited to form edge contacts. Layer thickness was verified using a Bruker Dimension FastScan AFM in tapping mode.

### Electrical measurements

We performed low-frequency measurement (excitation frequency ∼13.373 Hz) and standard Hall measurement using the TeslatronPT system (Oxford Instrument). The DC current of 100 nA was applied to avoid Joule heating. Local current annealing was performed with 1 mA/μm of DC current at 300 K for 120 min.

## Supplementary Material

nwad279_Supplemental_File

## References

[1] Lundstrom MS , Alam MA. Moore's law: the journey ahead. Science 2022; 378: 722–3. 10.1126/science.ade2191 36395227

[2] Bresniker K , Dukes S, Garner M et al. International roadmap for devices and systems. New Jersey, USA: Institute of Electrical and Electronic Engineer, 2022, 3–31.

[3] Schwierz F . Graphene transistors. Nat Nanotechnol 2010; 5: 487–96. 10.1038/nnano.2010.89 20512128

[4] Prasad C , Ramey S, Jiang L. Self-heating in advanced CMOS technologies. IEEE International Reliability Physics Symposium (IRPS). Piscataway, NJ, 2017, 6A–4.1–7.

[5] Roy K , Mukhopadhyay S, Mahmoodi-Meimand H. Leakage current mechanisms and leakage reduction techniques in deep-submicrometer CMOS circuits. Proc IEEE 2003; 91: 305–27. 10.1109/JPROC.2002.808156

[6] Snowden CM . Introduction to Semiconductor Device Modelling. Singapore: World Scientific Publishing, 1998, 14–36.

[7] Schwierz F . Graphene transistors: status, prospects, and problems. Proc IEEE 2013; 101: 1567–84. 10.1109/JPROC.2013.2257633

[8] Liu Y , Shivananju BN, Wang YS et al. Highly efficient and air-stable infrared photodetector based on 2D layered graphene-black phosphorus heterostructure. ACS Appl Mater Interfaces 2017; 9: 36137–45. 10.1021/acsami.7b09889 28948769

[9] Kang J , Jariwala D, Ryder CR et al. Probing out-of-plane charge transport in black phosphorus with graphene-contacted vertical field-effect transistors. Nano Lett 2016; 16: 2580–5. 10.1021/acs.nanolett.6b00144 26950174

[10] Dean CR , Young AF, Meric I et al. Boron nitride substrates for high-quality graphene electronics. Nat Nanotechnol 2010; 5: 722–6. 10.1038/nnano.2010.172 20729834

[11] Bolotin KI , Sikes KJ, Jiang Z et al. Ultrahigh electron mobility in suspended graphene. Solid State Commun 2008; 146: 351–5. 10.1016/j.ssc.2008.02.024

[12] Du X , Skachko I, Barker A et al. Approaching ballistic transport in suspended graphene. Nat Nanotechnol 2008; 3: 491–5. 10.1038/nnano.2008.199 18685637

[13] Wang L , Meric I, Huang PY et al. One-dimensional electrical contact to a two-dimensional material. Science 2013; 342: 614–7. 10.1126/science.1244358 24179223

[14] Robinson JA , LaBella M, Zhu M et al. Contacting graphene. Appl Phys Lett 2011; 98: 053103. 10.1063/1.3549183

[15] Li XL , Wang XR, Zhang L et al. Chemically derived, ultrasmooth graphene nanoribbon semiconductors. Science 2008; 319: 1229–32. 10.1126/science.1150878 18218865

[16] Son YW , Cohen ML, Louie SG. Energy gaps in graphene nanoribbons. Phys Rev Lett 2006; 97: 216803. 10.1103/PhysRevLett.97.216803 17155765

[17] Chen ZH , Lin YM, Rooks MJ et al. Graphene nano-ribbon electronics. Physica E Low Dimens Syst Nanostruct 2007; 40: 228–32. 10.1016/j.physe.2007.06.020

[18] Jiao LY , Zhang L, Wang XR et al. Narrow graphene nanoribbons from carbon nanotubes. Nature 2009; 458: 877–80. 10.1038/nature07919 19370031

[19] Zhang YB , Tang TT, Girit C et al. Direct observation of a widely tunable bandgap in bilayer graphene. Nature 2009; 459: 820–3. 10.1038/nature08105 19516337

[20] Sui MQ , Chen GR, Ma LG et al. Gate-tunable topological valley transport in bilayer graphene. Nat Phys 2015; 11: 1027–31. 10.1038/nphys3485

[21] Castro Neto AH , Guinea F, Peres NMR et al. The electronic properties of graphene. Rev Mod Phys 2009; 81: 109–62. 10.1103/RevModPhys.81.109

[22] Wang YY , Ni ZY, Liu QH et al. All-metallic vertical transistors based on stacked Dirac materials. Adv Funct Mater 2015; 25: 68–77. 10.1002/adfm.201402904

[23] Wang JY , Zhao RQ, Yang MM et al. Inverse relationship between carrier mobility and bandgap in graphene. J Chem Phys 2013; 138: 084701. 10.1063/1.4792142 23464166

[24] Liao L , Bai JW, Cheng R et al. Top-gated graphene nanoribbon transistors with ultrathin high-k dielectrics. Nano Lett 2010; 10: 1917–21. 10.1021/nl100840z 20380441 PMC2965644

[25] Mao JH , Milovanovic SP, Andelkovic M et al. Evidence of flat bands and correlated states in buckled graphene superlattices. Nature 2020; 584: 215–20. 10.1038/s41586-020-2567-3 32788735

[26] Levy N , Burke SA, Meaker KL et al. Strain-induced pseudo-magnetic fields greater than 300 tesla in graphene nanobubbles. Science 2010; 329: 544–7. 10.1126/science.1191700 20671183

[27] Nigge P , Qu AC, Lantagne-Hurtubise É et al. Room temperature strain-induced Landau levels in graphene on a wafer-scale platform. Sci Adv 2019; 5: eaaw5593. 10.1126/sciadv.aaw5593 31723598 PMC6839937

[28] Gui G , Li J, Zhong JX. Band structure engineering of graphene by strain: first-principles calculations. Phys Rev B 2008; 78: 075435. 10.1103/PhysRevB.78.075435

[29] Si C , Sun ZM, Liu F. Strain engineering of graphene: a review. Nanoscale 2016; 8: 3207–17. 10.1039/C5NR07755A 26796960

[30] Lee JK , Yamazaki S, Yun H et al. Modification of electrical properties of graphene by substrate-induced nanomodulation. Nano Lett 2013; 13: 3494–500. 10.1021/nl400827p 23848516

[31] Kim S , Konar A, Hwang WS et al. High-mobility and low-power thin-film transistors based on multilayer MoS_2_ crystals. Nat Commun 2012; 3: 1011. 10.1038/ncomms2018 22910357

[32] Tayari V , Hemsworth N, Fakih I et al. Two-dimensional magnetotransport in a black phosphorus naked quantum well. Nat Commun 2015; 6: 7702. 10.1038/ncomms8702 26151889 PMC4506510

[33] Tran S , Yang JW, Gillgren N et al. Surface transport and quantum Hall effect in ambipolar black phosphorus double quantum wells. Sci Adv 2017; 3: e1603179. 10.1126/sciadv.1603179 28630916 PMC5457033

[34] Luo F , Fan YS, Peng G et al. Graphene thermal emitter with enhanced joule heating and localized light emission in air. ACS Photon 2019; 6: 2117–25. 10.1021/acsphotonics.9b00667

[35] Bae MH , Ong ZY, Estrada D et al. Imaging, simulation, and electrostatic control of power dissipation in graphene devices. Nano Lett 2010; 10: 4787–93. 10.1021/nl1011596 20521804

[36] O’Farrell ECT , Tan JY, Yeo Y et al. Rashba interaction and local magnetic moments in a graphene-BN heterostructure intercalated with Au. Phys Rev Lett 2016; 117: 076603. 10.1103/PhysRevLett.117.076603 27563982

[37] Liu YP , Rodrigues JNB, Luo YZ et al. Tailoring sample-wide pseudo-magnetic fields on a graphene-black phosphorus heterostructure. Nat Nanotechnol 2018; 13: 828–34. 10.1038/s41565-018-0178-z 29941889

[38] Eckmann A , Felten A, Mishchenko A et al. Probing the nature of defects in graphene by Raman spectroscopy. Nano Lett 2012; 12: 3925–30. 10.1021/nl300901a 22764888

[39] Casiraghi C , Hartschuh A, Qian H et al. Raman spectroscopy of graphene edges. Nano Lett 2009; 9: 1433–41. 10.1021/nl8032697 19290608

[40] Ferrari AC , Basko DM. Raman spectroscopy as a versatile tool for studying the properties of graphene. Nat Nanotechnol 2013; 8: 235–46. 10.1038/nnano.2013.46 23552117

[41] Neumann C , Reichardt S, Venezuela P et al. Raman spectroscopy as probe of nanometre-scale strain variations in graphene. Nat Commun 2015; 6: 8429. 10.1038/ncomms9429 26416349 PMC4598719

[42] Gadelha AC , Ohlberg DAA, Rabelo C et al. Localization of lattice dynamics in low-angle twisted bilayer graphene. Nature 2021; 590: 405–9. 10.1038/s41586-021-03252-5 33597759

[43] Bustamante JV , Wu NJ, Fermon C et al. Detection of graphene's divergent orbital diamagnetism at the Dirac point. Science 2021; 374: 1399–402. 10.1126/science.abf9396 34882473

[44] Wang MK , Zhu J, Zi Y et al. Functional two-dimensional black phosphorus nanostructures towards next-generation devices. J Mater Chem A 2021; 9: 12433–73. 10.1039/D1TA02027G

[45] Hu HG , Shi Z, Khan K et al. Recent advances in doping engineering of black phosphorus. J Mater Chem A 2020; 8: 5421–41. 10.1039/D0TA00416B

[46] Low T , Roldán R, Wang H et al. Plasmons and screening in monolayer and multilayer black phosphorus. Phys Rev Lett 2014; 113: 106802. 10.1103/PhysRevLett.113.106802 25238376

[47] Villegas CEP , Rocha AR, Marini A. Anomalous temperature dependence of the band gap in black phosphorus. Nano Lett 2016; 16: 5095–101. 10.1021/acs.nanolett.6b02035 27428304

[48] Ng NK , Xiang D, Suwardi A et al. Improving carrier mobility in two-dimensional semiconductors with rippled materials. Nat Electron 2022; 5: 489–96. 10.1038/s41928-022-00777-z

[49] Poncé S , Margine ER, Giustino F. Towards predictive many-body calculations of phonon-limited carrier mobilities in semiconductors. Phys Rev B 2018; 97: 121201. 10.1103/PhysRevB.97.121201

[50] Purdie DG , Pugno NM, Taniguchi T et al. Cleaning interfaces in layered materials heterostructures. Nat Commun 2018; 9: 5387. 10.1038/s41467-018-07558-3 30568160 PMC6300598

[51] Stenger I , Schué L, Boukhicha M et al. Low frequency Raman spectroscopy of few-atomic-layer thick hBN crystals. 2D Mater 2017; 4: 031003. 10.1088/2053-1583/aa77d4

