# Supplementary material for: Graphene binding on black phosphorus enables high on/off ratios and mobility
Source: Natl Sci Rev. 2023 Nov 3;11(2):nwad279. doi: 10.1093/nsr/nwad279 (PMC10776355; doi:10.1093/nsr/nwad279)
Supplement: nwad279_Supplemental_File [file nwad279_supplemental_file.pdf]

Supplementary Information

Graphene binding on black phosphorus enables high on/off ratios and mobility

Fanrong Lin[1,†], Zhonghan Cao[2,†], Feiping Xiao[3], Jiawei Liu[2], Jiabin Qiao[4], Minmin Xue[1], Zhili Hu[1], Ying Liu[1], Huan Lu[1], Zhuhua Zhang[1], Jens Martin[5], Qingjun Tong[3,*], Wanlin Guo[1,*] and Yanpeng Liu[1,*]

[1]Key Laboratory for Intelligent Nano Materials and Devices of Ministry of Education, State Key Laboratory of Mechanics and Control of Mechanical Structures, and Institute for Frontier Science, Nanjing University of Aeronautics and Astronautics, Nanjing 210016, China
[2]Centre for Advanced 2D Materials, National University of Singapore, 6 Science Drive 2, 117546, Singapore
[3]School of Physics and Electronics, Hunan University, Changsha 410082, China
[4]Centre for Quantum Physics, Key Laboratory of Advanced Optoelectronic Quantum Architecture and Measurement, School of Physics, Beijing Institute of Technology, Beijing 100081, China
[5]Leibniz Institute für Kristallzüchtung, Max-Born-Strasse 2, Berlin, 12489, Germany
*Corresponding author. Email: wlguo@nuaa.edu.cn; chmliuyp@nuaa.edu.cn; tongqj@hnu.edu.cn

Contents

1. Current annealing and Raman characterizations S3

1.1. Local current annealing S3

1.2. Raman spectrum of graphene-based sample S4

1.3. Electronic localization in strained graphene S7

2. Electronic structure and properties of graphene-based transistor S9

2.1. First-principles calculation of 0°-Gr/BP S9

2.2. Theoretical modeling of charge accumulation in BP S11

2.3. Electronic properties of Gr/BP sample S12

2.4. Charge transfer quantum Hall effect S16

2.5. Anisotropic charge mobility S18

2.6. Landauer-Büttiker transmissions S23

2.7. Single wave propagation through reflective interface S25

2.8. Gate tunable nonlocal results S27

2.9. Magneto response of inter-junction resistance S29

2.10. Differential conductance in multi-terminal graphene-based transistor S31

3. Parallel propagation S33

3.1. Evidences of parallel propagation S33

3.2. Theoretical simulation of longitudinal $V_{51,32}$ S35

3.3. Theoretical calculation of transverse $V_{51,73}$ S35

3.4. Simulating the negative $V_{51,32}$ under magnetic fields S36

4. Temperature-dependence resistance in pristine graphene S38

5. Performance summary of assorted field effect transistors S40

6. Reference S41

1. **Current annealing and Raman characterizations**

1.1. **Local current annealing**

Before current annealing, the four-terminal resistance $R_{51,32}$ (resistance between electrodes 3 and 2, current injecting from electrode 5 to electrode 1) as function of V_{bg} measured at T = 1.5 K is shown in Figure S1a. Current annealing was performed by applying 1 mA/μm in between electrodes 1 and 3 for 120 min at room temperature. The Joule heat was recognized to distort the local lattice and even introduce local disorder [S1,S2]. For intuitive, the current density distribution is sketched in Figure S1b and the red area is proposed to undergo a higher current density as well as Joule heat.

After heating, BP tends to thermally expand along the out-of-plane direction[S3], whereas the thermal coefficient of graphene is negligible due to the ultra-high thermal conductivity[S4]. Therefore, the Gr/BP interface experiencing thermal annealing would definitely undergo out-of-plane deformation. As a result, the local lattice distortion and possible non-uniform moiré disorder arise in the red region, forming a strain junction in between un-annealed and annealed graphene.

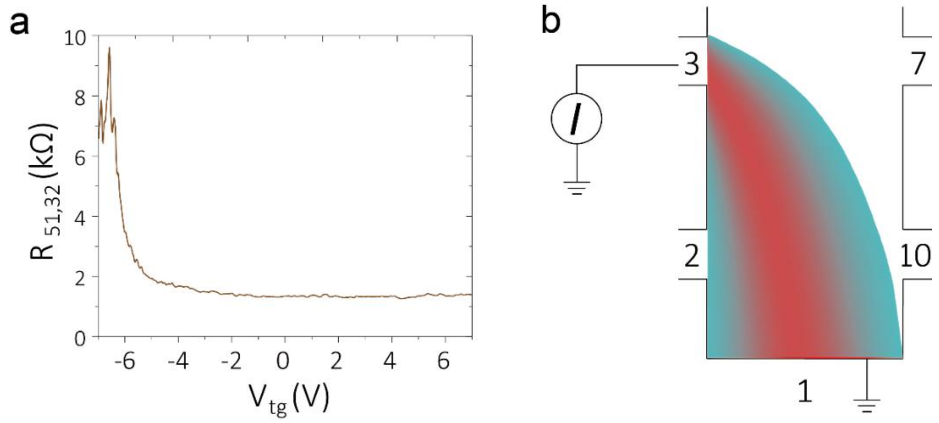

Figure S1 | **a**, Four terminal resistance $R_{51,32}$ as function of top-gate voltages before current annealing at V_{bg} = 0 V, T = 1.5 K and B = 0 T. **b**, Schematic of Joule heat distribution during current annealing. Red refers to the region with higher current density and Joule heat.

1.2. **Raman spectrum of graphene-based sample**

Interlayer coupling between Gr and BP heterostructure can be nicely resolved by Raman spectrum that characterizes the phonon energy at $\Gamma = 0$. Figure S2a-2d show Raman signals of 0 °-1L Gr/BP (twist angle ~ 0°), 2 °-4L Gr/BP, 5 °- 1L Gr/BP, and 10 °-1L Gr/BP samples. As can be seen in Figure S2a, the Raman spectrum of annealed Gr differs dramatically. Comparing the Raman signals at different locations of this sample, strained Gr shows broadened G and 2D peaks and extra peaks at 1340.3 cm^{-1}, 1615.4 cm^{-1}, and 2932.7 cm^{-1}, corresponding to D, D' and (D+D') peaks of graphene.

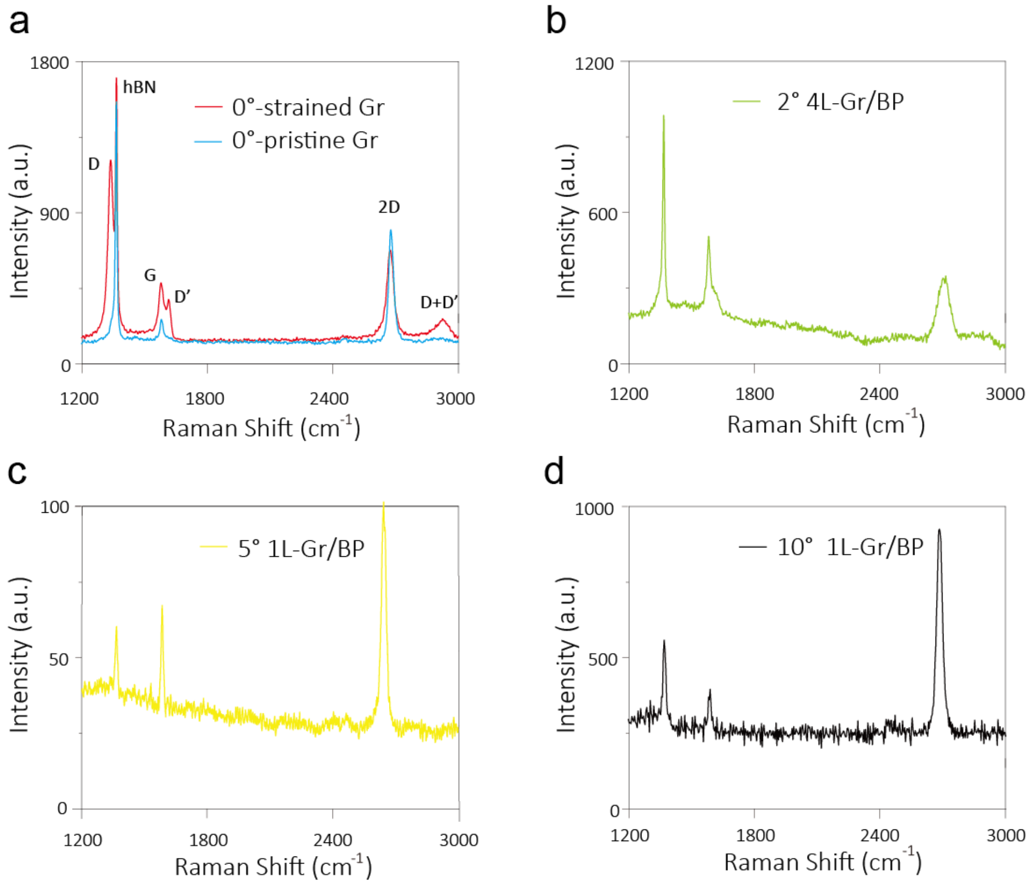

Figure S2 | Raman characterization of Gr/BP stacks with different twisted angle. a, Red and blue lines correspond to strained and unstrained Gr in 0 °-1 layer (1L) Gr/BP stack. **b-d**, green, yellow, black corresponds to the 2 °-4L-graphene/BP, 5 °-1L-graphene/BP and 10 °-1L-graphene/BP, respectively.

We extracted the full width at half maximum (FWHM) of G peaks (Γ_G) and 2D peaks (Γ_{2D}) at different spatial locations. The plots of peak position (ω) and FWHM of G and 2D peaks are shown in Figure S3a-3c. Red and blue dots denote the data taken at

annealed and un-annealed Gr, respectively. Notably, the extracted Γ_G and Γ_{2D} in annealed Gr are significantly larger than that from un-annealed Gr. We temporarily argue that the peak broadening may originate from the appearance of nanometer-scaled strain field. Since the diameter of laser spot in our experiments is ~1 μm, the collected Raman spectra could be referred as the averaged signal of strained graphene at nanometer scale. Consequently, the integrated peaks manifest itself as a broadened G and 2D peaks[S5]

We next discussed the strain origin of D, D', and (D+D')-peaks. The origin of D peak could be referred as the electron/hole backscattering induced by defects or armchair edges that activate the double resonance process. The detailed process (left panel, Figure S3d) is as follow: i) electron-hole pair generation via external laser; ii) intervalley electron-phonon scatterings; iii) backscattering; iv) electron-hole recombination. Similarly, the D' peak is due to the intravalley scattering (middle panel, Figure S3d), while the (D+D')-peak is the synergistic of intervalley and intravalley scattering process. In our samples, the signals of the annealed and pristine Gr were collected from the same sheet while D (D', D+D') peaks were absent at the pristine Gr region. Therefore, the electron/hole backscattering originates from the strain field instead of defects nor armchair edges. Figure S3e shows the reciprocal space scheme of phonon-assisted backscattering process. Phonon wavevector $\boldsymbol{q}$ starts from one valley $(K + \boldsymbol{k'})$ and ends in the other valley. To achieve backscattering, phonon wavevector $\boldsymbol{q'}(= \boldsymbol{q} - \boldsymbol{KK'}$, where $\boldsymbol{KK'}$ is the quasi-momentum between graphene K and K' valleys) satisfies $\boldsymbol{q'} = -2\boldsymbol{k'}$, as shown in Figure S3e. In the strained graphene, the structural deformation of graphene localizes the charges manifested as flat pseudo-Landau levels (as shown in Figure 1i in the maintext). Figure S3f sketches the charge localization phenomenon with possible electron (hole) backscattering. With these information, we could argue that the graphene nanoripples, distorted BP top surface (Figure 1h) as well as moiré disorders are the main origins of the observed Raman features and differential transport conductance (Figure 1e).

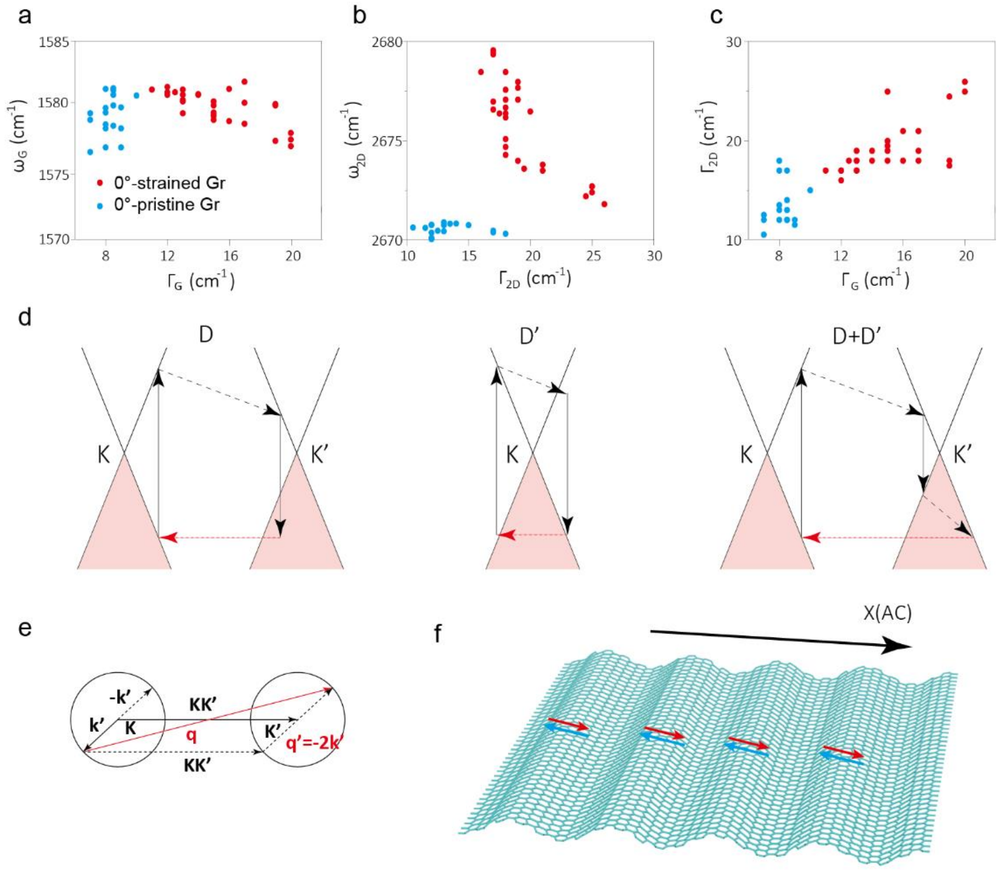

Figure S3 | Raman evidences of strain induced charge localization in Gr/BP. a, Relation of Raman shifts and FWHM of G peaks at multiple selected areas. **b**, Statistical Raman shifts and FWHM of 2D peaks. **c**, Representative FWHMs of graphene G and 2D peaks from two regions. **d**, Double-resonance process of D (left panel), D' (middle panel), D+D' (right panel) peaks. **e**, Reciprocal space of phonon-assisted backscattering. **f**, Schematic of charge localization in the strained graphene. Red and blue arrows denote the moving charges with strong back-scatterings.

1.3. **Electronic localization in strained graphene**

To probe the strain field in well-aligned (0°) Gr/BP sample and associated electronic properties, scanning tunneling microscope (STM, Scientaomicron) was performed on annealed Gr/BP surface in ultrahigh-vacuum condition (< 10^{-11} torr). The tungsten tip was pre-calibrated on the surface of Au (111) sample. The Gr/BP sample was annealed at 150 °C in ultrahigh vacuum (< 1 × 10^{-10} torr) for 10 hours[S8] All STM data were captured in constant-current mode at 77 K. The scanning tunneling spectrum (STS) were acquired with the assistance of external lock-in technique (SR830 Lock-In Amplifier, bias modulations of 5 - 10 mV at 763.1 Hz).

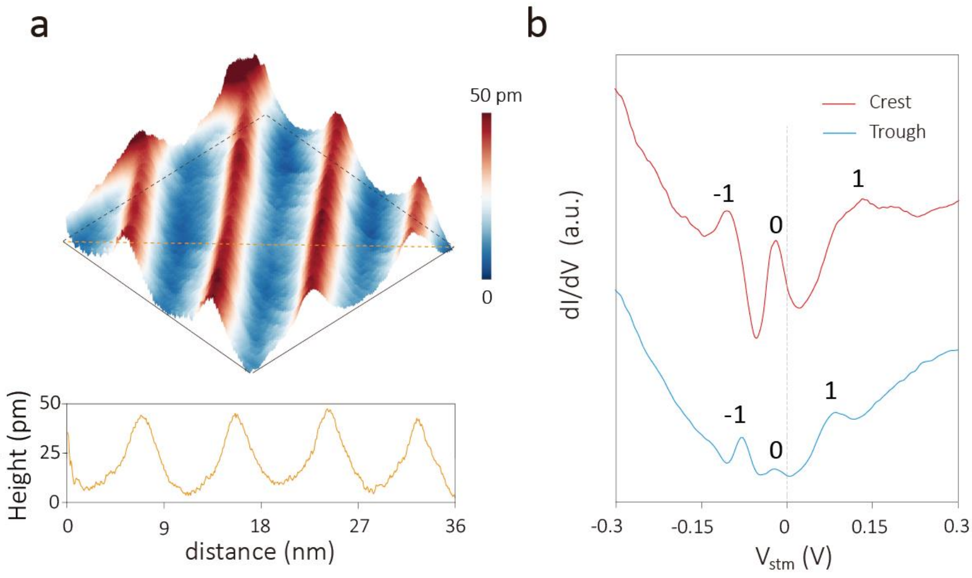

Figure S4 | STM/STS study of annealed Gr/BP sample. a, Surface topography (25 nm×25 nm) of quasi-1D Gr/BP moiré superlattice. **b**, STS data collected at the crest (red curve) and trough (blue curve) regions of Gr/BP sample.

Figure S4a shows the quasi-1D superlattice of Gr/BP sample with spatial periodicity ~8 nm. The subsequent STS was performed on the crest and trough regions as shown in Figure S4b. Notably, the arisen peaks are attributing to the pseudo-Landau levels with $N = 0$ and ±1. The pseudo magnetic field (PMF) was respectively estimated ~16 T and ~9 T for crest and trough regions according to the single-particle Landau quantization formula[S6]

$$E_N - E_{DP} = \mathrm{sgn}(N)\sqrt{2e\hbar v_F^2 B_{PMF}} \times \sqrt{|N|} \tag{S1}$$

where E_N and E_{DP} refer to the energy of Nth pseudo-Landau level and Dirac point. B_{PMF} is the pseudo-magnetic field and v_F refers to the Fermi velocity. The PMF intensity grows with decreasing twist angle θ resulting from stronger interlayer coupling. Consequently, the calculated bandwidth of the zero-energy flat-band heavily depends on the PMF intensity and moiré superlattice periodicity[S7].

According to the equation (3) and (4) in the maintext, with $\frac{e}{h} = 0.25 \times 10^{-5} \frac{1}{\mathrm{T{\cdot}\AA^2}}$ for moir épattern of L_x = 100 Å, $\omega_x = \frac{2\pi}{L_x}$, B = 10 T, $A_2 \sim 10\ \mathrm{T} \cdot \frac{L}{2\pi}$, we estimated the $\beta_2 = \frac{eA_2}{\hbar\omega_x} \sim 0.25$, $\beta_1 \sim \frac{L_y}{L_x}\frac{A_1}{A_2}\beta_2$. For $\beta_2 \sim 2$, the pseudo magnetic field is estimated to be about 80 T. With these information, the band structure and density of states under pseudo-magnetic field $\beta_2 \sim 2$ and ~ 8 were simulated (Figure S5). For larger strain field, the pseudo-Landau levels become flattened and their energy difference tends to be increased. These theoretical calculations are consistent with STM and differential transport conductance. The electronic structure of Gr/BP heterostructure is heavily depended on the twist angle. The low-energy states of graphene are found to become flatter and denser at smaller twist angle θ, promoting the switching behavior in the concurrence of satisfied mobility.

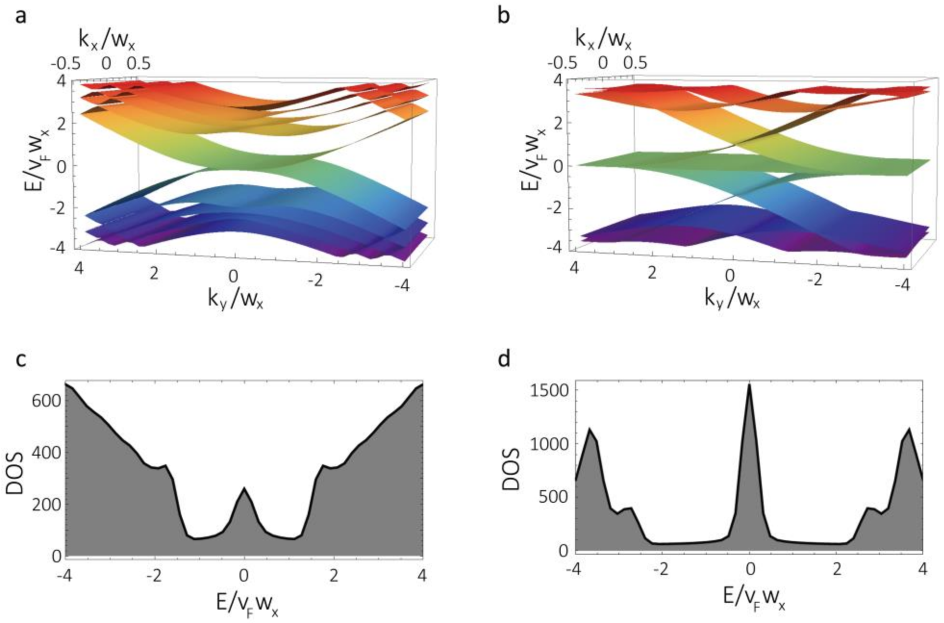

Figure S5 | Minibands for graphene in moiré-modulated pseudo-magnetic field with periodicity $L_x = 5L_y$. Ten minibands near zero energy are shown. **a** and **b**, Calculated band structures of graphene in a moiré-induced pseudo-magnetic field by adopting $\beta_2 = 5\beta_1 = 2$ and $\beta_2 = 5\beta_1 = 8$, respectively. **c**, Corresponding density of states of graphene for $\beta_2 = 5\beta_1 = 2$. **d**, Simulated density of states of graphene by employing $\beta_2 = 5\beta_1 = 8$.

2. Electronic structure and properties of graphene-based transistor

2.1. First-principles calculation of 0°-Gr/BP

To simulate Gr monolayer on BP multilayer, we adopted the configuration of monolayer graphene and four-layer BP, as shown in Figure S6a-6c. Figure S6d shows the calculated electronic band structure. For BP multilayer, AB stacking order (with an interlayer shift of $b/2$ along the zigzag direction) is selected since it is the most energy-favorable configuration.

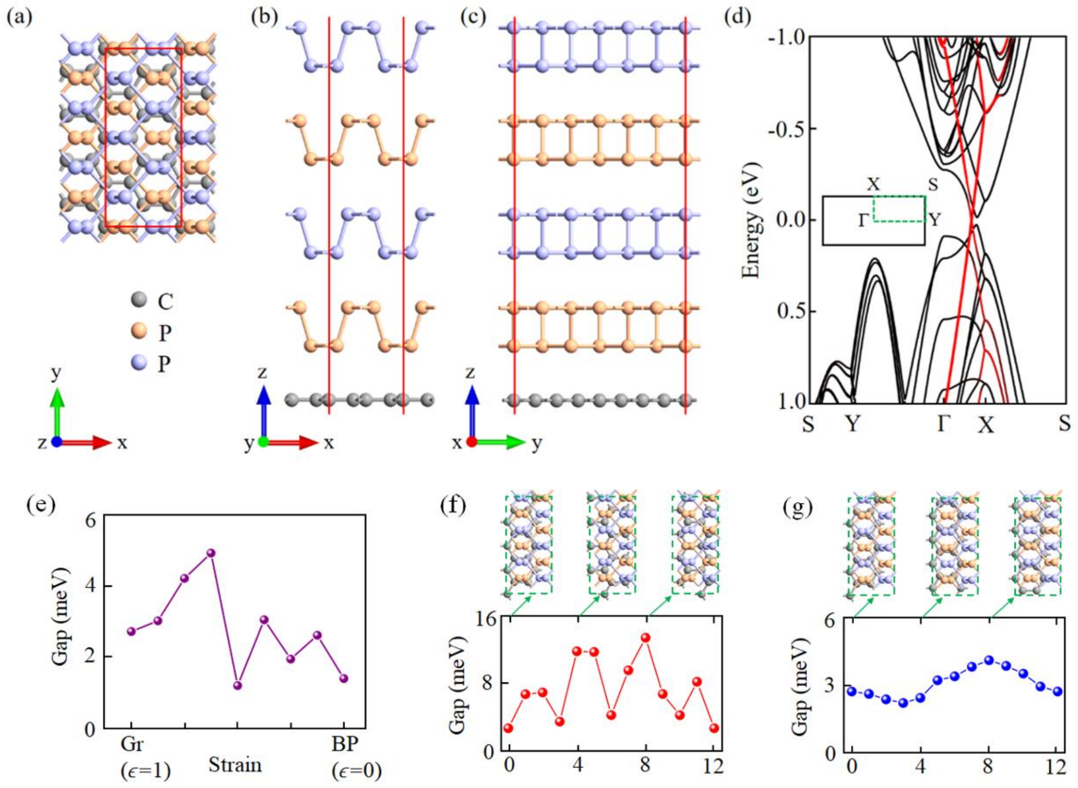

Figure S6 | Electronic structure of 0°- Gr/BP sample. Top (**a**) and side (**b**, **c**) views of atomic structures of commensurate Gr/BP sample. The red rectangle denotes a supercell (4.26 × 9.84 Å). **d**, Calculated electronic band structure of monolayer graphene on four-layer BP. **e**, First-principles calculated bandgap of graphene/BP as a function of strain. **f**, **g**, Calculated band gap of graphene when stacked on BP for different stacking orders. The schematics of typical stacking orders are shown atop.

Departing from the optimized BP multilayer, we applied compressive strains along the armchair and zigzag directions and defined the strained lattice constants as $a'_p = a_p - \epsilon(a_p - a_g)$ and $b'_p = b_p - \epsilon(b_p - b_g)$ with a single compressive strain parameter ϵ. $\epsilon = 0$ corresponds to the case of pristine lattice constants of phosphorene, while $\epsilon = 1$

corresponds to the scenario of graphene lattice constants. Figure S7a-7c show the calculated strain-dependent band structures and corresponding density of states (DOS). During increasing the compressive strain strength, the band gap decreases gradually and finally closes. In accordance to the first-principles calculation of Gr/BP heterostructure, the bandgap value of in-plane strained graphene (strain is from 0 to 7.5%) is about 1 ~ 5 meV (Figure S6e). To estimate the stacking effect, we further calculated the band structure via shifting graphene with respect to BP layer (Figure S6f and S6g). It is found that the sliding of Gr along the armchair direction of Gr (same as BP) varies strongly the band gap of graphene while sliding along zigzag direction has slight impact of graphene bandgap.

With respect to graphene under sufficient in-plane strains, the first-principles calculation shows the bandgap of distorted graphene approaches about 3 meV. The strain fields due to the lattice distortion decreases graphene Fermi velocity and eventually deliveries multiple flat pseudo-Landau levels (Figure S4, S5).

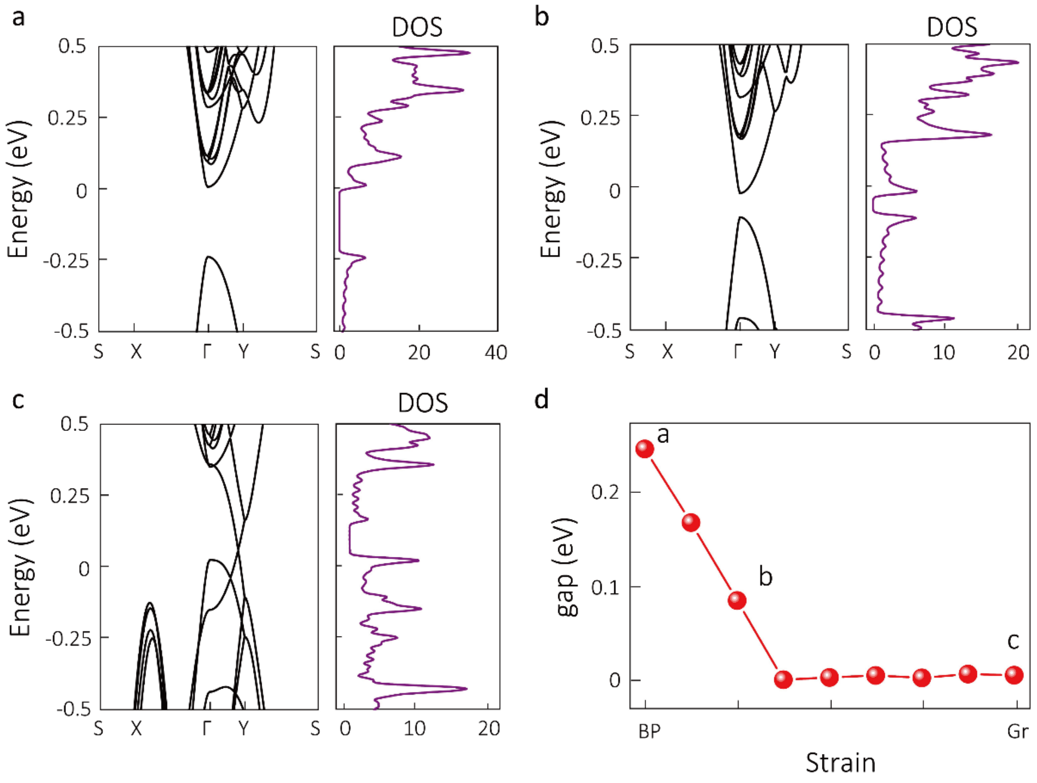

Figure S7 | Electronic properties of strained BP. a-c, The band structures and DOS of four-layer BP at different compressive strains $\epsilon = 0$ (**a**), 0.25 (**b**) and 1 (**c**), respectively. **d**, The plots of calculated BP bandgap versus compressive strain parameter ϵ.

2.2. **Theoretical modeling of charge accumulation in BP**

We employed first-principle calculations and model extrapolation to prove the charge accumulation of ten-layer BP in the presence of external electric fields. The layer-resolved free-carrier-density distribution was obtained by integrating the local density of states from the Fermi level to the valence band maximum (conduction band minimum) for holes (electrons)[S9,S10]. The calculations work well up to a certain value of electric fields, especially when the band gap closes. We then extrapolated the high-field charge accumulations from calculated low-field ones using the Airy function $\varphi(z) = V \times Ai(\frac{z}{L} + a_1)$, where $a_1 = 2.338$ is the first zero of Airy function. V and L are two fitting parameters and the employed parameters are $V = (4.932, 6.303, 7.673, 9.043, 10.413) \times 10^{20}$ cm^{-3} and $L/d = (2.5, 2, 1.5, 1, 0.5)$ with d the interlayer distance for applied field $E = (0.1, 0.2, 0.3, 0.4, 0.5)$ V/nm, respectively. Figure S8 shows the free carrier accumulation at the sample surface at higher external electric fields, supporting our observations of surface propagation mode in graphene-based transistor.

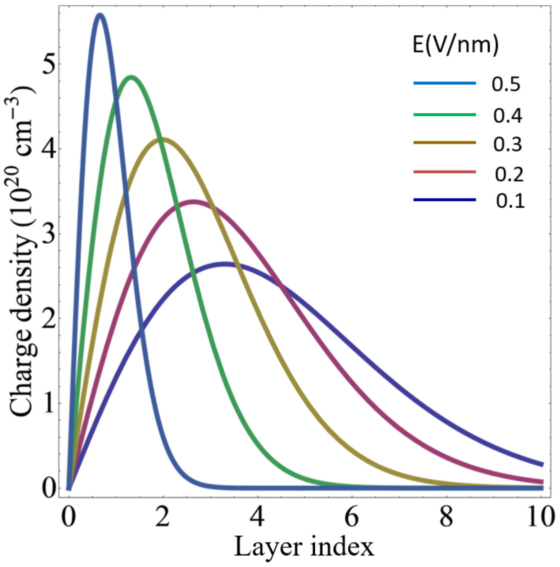

Figure S8 | Charge distribution of a hole-doped ten-layer BP subjected to electric fields. Charge distribution at high electric fields (> 0.3 V/nm) are obtained by extrapolation from the first-principles calculated carrier density at low fields.

2.3. **Electronic properties of Gr/BP sample**

The charge transport at Gr/BP interface can be divided into two parts, mobile charges in graphene and BP top channels. The charged thickness is determined by the depletion thickness of BP (W_D, Figure S9). The charge neutrality of BP requires $Q_M = -eN_{3D}W_D$, where Q_M is charges per unit area on the back-gate and N_{3D} is bulk carrier density of doped BP ($N_{3D} = N_{2D}/d$). An estimated thickness of depletion layer as a function of V_{bg} is

$$W_D = \frac{\epsilon_{BP}}{C_{bg}}\left(\sqrt{1+\frac{2\epsilon_{bg}^2 V_{bg}}{eN_{3D}\epsilon_{BP}d^2}}-1\right) \quad \text{(S2)}$$

where C_{bg}, ϵ_{BP}, ϵ_{bg}, d refer to the capacitance of back-gate, dielectric constant of BP, dielectric constant and dielectric thickness of substrate (including hBN and SiO_2)[S11]. Typically, for $N_{3D} = 1\times10^{18}$ cm^{-3}, $\epsilon_{BP} = 8\epsilon_0$, $\epsilon_{bg} = 4\epsilon_0$, the numerical estimation W_D is ~ 22 nm at $V_{bg} = 30$ V, comparable with the BP thickness in experimental study.

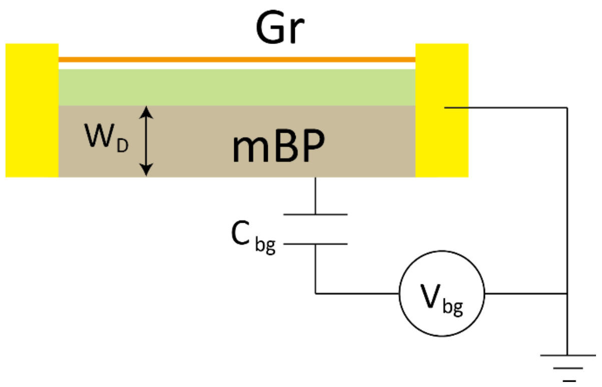

Figure S9 | Schematic of depleted BP. W_D is the depletion layer thickness under specific V_{bg}.

The BP bandgap varies its thickness from 2 eV for monolayer to 0.3 eV for bulk. In our experiments, the lower limit of BP thickness is examined to be ~6 nm to minimize the charge exchange in between top Gr and bottom BP channels. For the upper limit, the charges tend to transport within the BP top channels once BP is thicker than the depletion thickness (~30nm), extremely diminishing the on/off ratio and mobility. Therefore, we believe that BP flakes with thickness ranging from ~6 to 30 nm is desirable in graphene/BP transistors here.

Experimentally, the robust Dirac-peak evolution as a function of V_{tg} (Figure 2d) reveals

the dominant signals from graphene layer. To unravel the magneto-transport, Figure S10a-10b show the non-quantized (a) and quantized (b) Hall carrier density as a function of V_{tg} for pristine graphene. At T = 120 K and B = 2 T, the transport behavior remains in the classical regime. The linear correlation of Hall carrier density (Figure S10a, converted from the Hall voltage according to $n_{\mathrm{Hall}} = \frac{BI_{51}}{eV_{51,46}}$) and varying V_{tg} indicates a capacitive coupling with top-gate voltages (C_t). Taking consideration of constant geometric capacitance from top hBN dielectric (C_{hBN}), the capacity with respect to the graphene (C_t) remains constant but with electron-hole asymmetry, presumably ascribed to interlayer charge transfer and BP surface states.

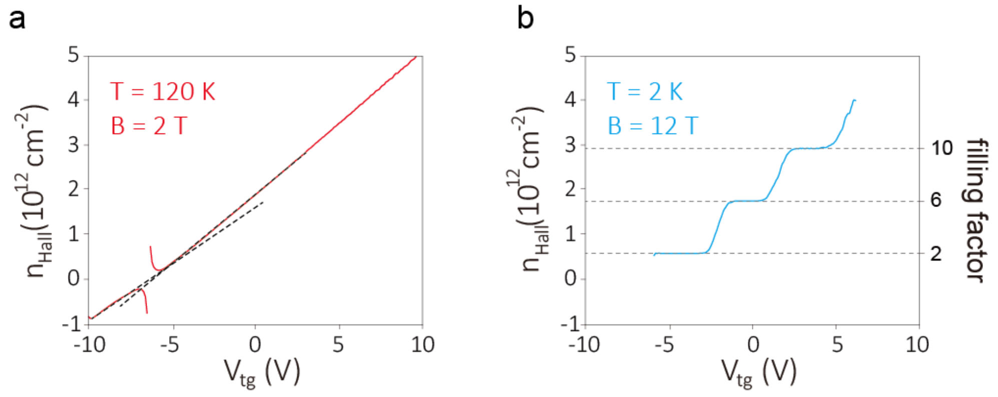

Figure S10 | Hall effect of pristine graphene on BP. **a**, Hall signal $n_{\mathrm{Hall}} = \frac{IB}{eV_{\mathrm{Hall}}}$ at varying top-gate voltages at T = 120 K and B = 2 T. Black dashed lines mark the linear Hall signals. **b**, Quantum Hall effect of pristine graphene on BP at T = 2 K and B = 12 T.

To further disclose the charge transport behavior at Gr/BP interface, a magnetic field of 12T was introduced to explore the quantum Hall (QH) effect (Figure S10b). The quantized Hall plateaus (filling factors v = 2, 6, 10) are fairly robust, evidencing that the dominant charge comes from graphene rather than BP layer.

Figure S11a shows the Shubnikov-de Haas (SdH) oscillation at high mobility graphene by measuring V_{xx} as function of magnetic fields (B) and back-gate voltages at V_{tg} = -1 V and T = 1.5 K. Two main features are identified. Firstly, the inter-junction transmission in between the annealed and pristine graphene remains 0 at $V_{bg} \geq 0$ V despite the applied magnetic fields, whereas the transmission raises for $V_{bg} < 0$ V due to the activated BP bottom channel, supporting the parallel conduction model. Secondly,

the activated V_{xx} shows the SdH oscillation and the QH states (yellow dashed lines). To confirm the SdH oscillation, the extracted filling factor v as function of 1/B (Figure S11b) generates a linear oscillation pattern. As a result, the Hall carrier density n_{Hall} = 1.5×10^{12} cm^{-2} at V_{tg} = -1 V could be extracted to be the same value at T = 120 K, indicating a thermally invariant graphene capacitance, in consistence with pristine graphene (Figure S10). It is worthy to mention that the negative V_{xx} at B > 10 T originates from the Hall effect of BP bottom channel as mentioned in the maintext.

The interlayer charge transfer leads to the Fermi-level pinning when coupling semiconductors with metals or semimetals. Therefore, coupling graphene with BP not only broadens the QH plateau width, but also generates an electron-hole asymmetric graphene capacitance C_t.

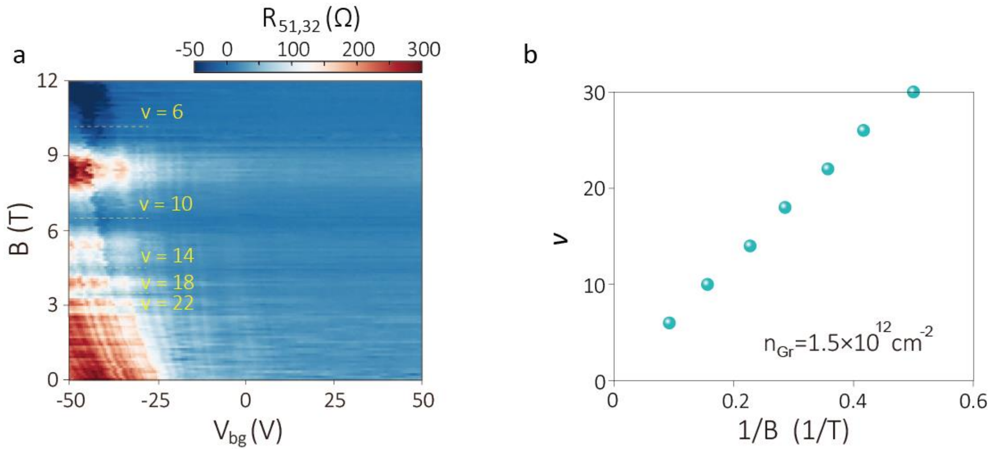

Figure S11 | Magneto dependence of graphene-based device. a, Resistance between electrodes 3 and 2 ($R_{51,32} = V_{xx}/I_{51}$) under 10 nA ($I_{51}$) current bias evolving with magnetic fields and back-gate voltages (T = 1.5 K, V_{tg} = -1 V). Yellow dashed lines mark different quantum Hall states. **b**, Extracted linear dependence of filling factor v with respect to 1/B.

The culmination of these findings suggests that the uppermost layer of BP sustains the charges in an electrostatic equilibrium. For the strain-free Gr/BP region, the charges primarily inhabit in the BP valence band. While for the strained region, the interfacial strain field also impinges BP topmost layers regions, modifies its local electronic band structure (Figure S7) and results in a strong localization of charges. Despite the existence of a reflective interface within topmost BP layer, charge propagation through this interface appears to be inhibited. This is largely attributable to the localization of

charges within strained regions of BP and the decreased carrier density that becomes evident with increasing depth into the BP. These observations suggest the negligible impact of upper BP layers on the overall switching performance.

2.4. **Charge transfer quantum Hall effect**

In addition to aforementioned electron-hole asymmetric capacitance and anisotropic charge mobility, interlayer charge transfer is also involved into the quantum Hall effect as extra charge localization centers. The developed Hall resistance plateaus at QH states correspond to the filling of localization centers. In a clean graphene sample, the minimized disorder centers narrow the QH plateau. In our experiments, however, the QH plateaus broadens resulting from the interlayer charge transfer. Figure S12a-12b show the Landau Fan diagram of longitudinal resistance $R_{51,67}$ and Hall resistance $R_{51,64}$ (pristine Gr). The broadened QH plateau (manifested as top-gate voltages, ΔV_{tg} multiplying the graphene capacitance C_t) ~ 6×10^{11} cm^{-2} at $v = 6$, $B = 12$ T, similar to Gr on SiO_2 sample[S12-S14]. The plateau width increases linearly with elevated magnetic field for all measurable filling factors. To explain these phenomena, we divided the Gr/BP interfacial charges into two parts, mobile charges at Gr layer (n_{Gr}) and the localized surface charges residing in BP top layers (n_{BP}^{top}, charge transferred from graphene). Hence, the parallel capacitor model can be modified as

$$n_M = n_{Gr} + n_{BP}^{top} \tag{S3}$$

where n_M is gate-induced carrier density corresponding to the hBN geometric capacitance C_{hBN}. At low temperature, by varying Fermi energy across the consecutive Landau levels, the amount of transferred charge n_{BP}^{top} can be regarded as integrating the in-Landau gap density of states with the Landau level energy difference. In the Gr monolayer, Landau levels at each filling factors are expressed as

$$E_n = \mathrm{sgn}(n) v_F \sqrt{2e\hbar B|n|} \tag{S4}$$

where n, v_F are Landau index ($v = 4n+2$) and Fermi velocity of graphene, respectively. Therefore, the Landau gaps E_{n+1} - E_n depend on the magnetic fields and filling factors. The largest Landau gap at $v = 2$ provides the dense n_{BP}^{top} that requires top-gate to fill these states. In addition, the elevated magnetic field promotes the Landau gap and carrier densities into the QH states. The linear increment of n_{BP}^{top} with B suggests that

the amount of interlayer transferred charges grows with B. This B-dependent interlayer charge transfer is associated with the growing QH states density with B.

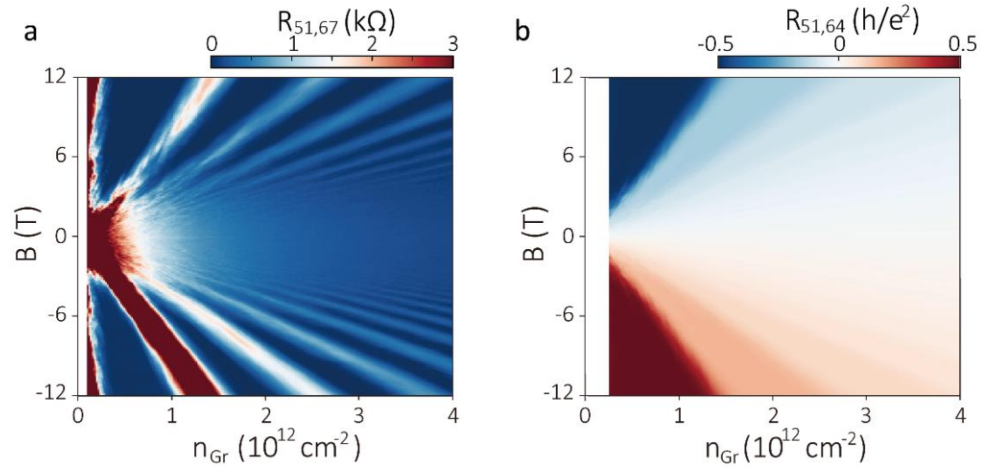

Figure S12 | Charge transfer quantum Hall effect of pristine Gr on BP. a, Landau fan diagram of longitudinal resistance $R_{51,67}$. **b**, Hall resistance $R_{51,64}$ of pristine Gr as function of magnetic field and carrier density. T = 1.5 K and V_{bg} = 50 V.

2.5. **Anisotropic charge mobility**

Graphene preserves C_{3v} symmetry that is isotropic in its basal plane. Incorporating graphene with anisotropic BP may break both the sublattice symmetry and the C_{3v} symmetry. The L-shape Hallbar device facilitated us to investigate the anisotropic transport of graphene on BP. Figure S13a shows the four-terminal measurements configuration of X-Hallbar (blue) and Y-Hallbar (red) at $T = 120$ K.

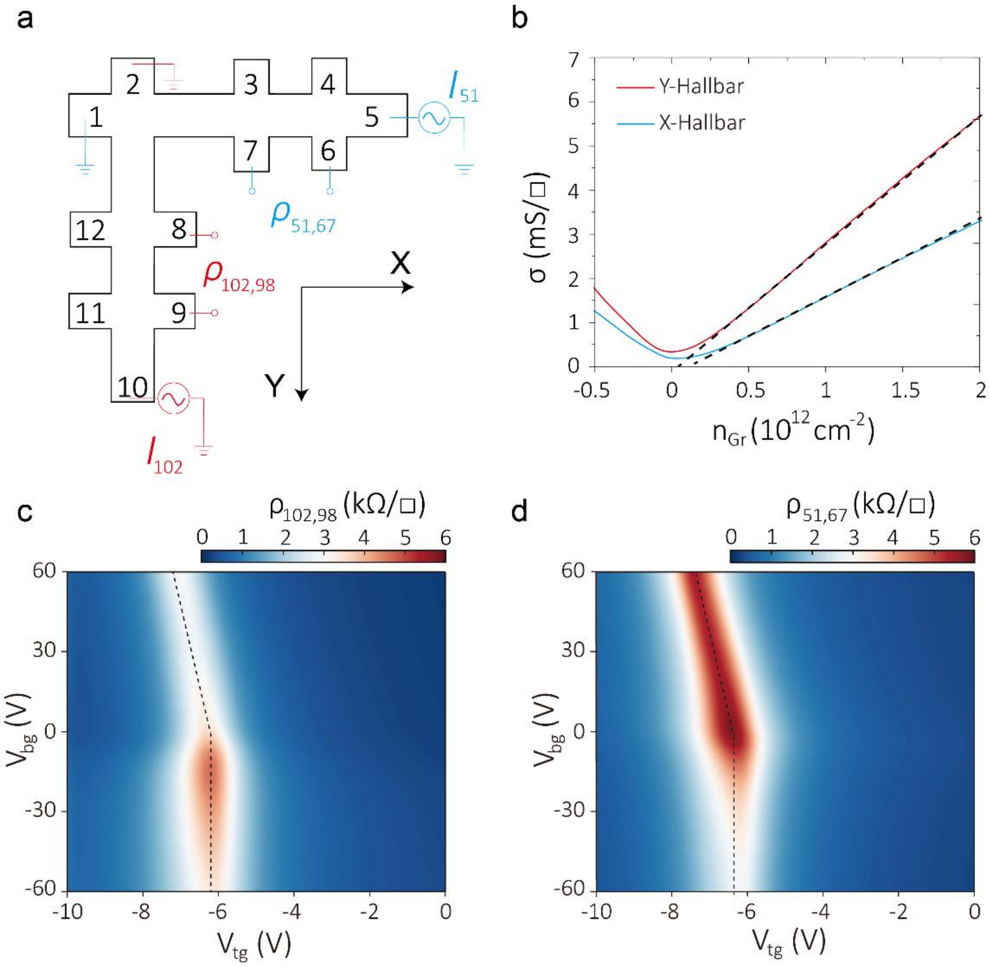

Figure S13 | Anisotropic transport in L-shape Hallbar device. **a**, Schematic of L-shape Hallbar device and four terminal measurements. **b**, Conductivities as a function of n_{Gr} along X and Y directions at $V_{bg} = 60$ V. **c**, **d**, Dual-gated maps of sheet resistance of Y-Hallbar (c) and X-Hallbar (d) at 0 T and 120 K. Black dashed lines mark the position of the graphene charge neutral point.

Figure S13b shows the anisotropic conductivities σ_X (blue) and σ_Y (red) as function of n_{Gr}, respectively. n_{Gr} is determined according to $n_{Gr} = C_t(V_{tg} - V_{DC})$ ($C_{t,e} = 3.1\times10^{11}$ cm^{-2}/V, at electron doped branch, $C_{t,h} = 2.2\times10^{11}$ cm^{-2}/V, at hole doped branch, $V_{DC} = -6.2$

V). For electron-doped region (black dashed lines in Figure S13b), two features are worthy to mention. Firstly, the X-Hallbar conductance is lower than Y-Hallbar conductance at same carrier densities, indicating the anisotropic transport in graphene-based transistor. Secondly, the sublinear σ - n_{Gr} for both X- and Y-Hallbars allows us to extract the electron mobility μ_x and μ_Y. The extracted μ_Y = 20,000 cm^2 V^{-1} s^{-1} is 1.6 times larger than μ_X = 12,000 cm^2 V^{-1} s^{-1}, suggesting anisotropic mobility in graphene after coupled with BP. Accordingly, the mean free path of charges can be calculated using $l_{\mathrm{mfp}} = (h/2e)\mu\sqrt{n_{\mathrm{Gr}}/\pi}$. The X(Y)-Hallbar mean free path at $n_{Gr} = 1\times10^{12}$ cm^{-2} is l_{mfp_X} = 140 nm (l_{mfp_Y} = 230 nm). This anisotropy is mainly assigned to the spatially orientation of moiré structure. The moiré pattern as well as the interlayer charge transfer could be regarded as "substrate roughness" that enhances the charge scatterings stronger along the X direction (moiré periodicity ~8 nm) than along Y direction (moiré periodicity ~1 nm). Specifically, the charge wavelength (λ) within the graphene (for instance, ~ 35 nm at a carrier density n = 10^{12} cm^{-2}) is more comparable with the superlattice periodicity in the zigzag orientation other than the one along the armchair direction. Consequently, the resultant wave interference along the armchair direction mitigates charge scatterings, whereas charge transport along the zigzag direction experiences more scatterings and exhibits a reduced carrier mobility.

In order to compare the graphene capacitances in X- and Y-Hallbars, we mapped the sheet resistance $\rho_{102,98}$ and $\rho_{51,67}$ as functions of V_{bg} and V_{tg} (Figure S13c-13d), respectively. Based on the afore-mentioned electronic structure, the carrier density of graphene (n_{Gr}) could be determined according to $n_{Gr} = \varepsilon_0\times(D_{top} - D_{bottom})$, where $D_{top} = -\varepsilon_{top}\times(V_{tg} - V_{tg0})/d_{top}$, $D_{bottom} = \varepsilon_{bottom}\times(V_{bg} - V_{bg0})/d_{bottom}$ are the top-gate and back-gate induced displacements fields (ϵ_{top} and d_{top} denote the effective dielectric constant and thickness of top hBN, ϵ_{bottom} and d_{bottom} represent effective dielectric constant and thickness of SiO_2, bottom hBN, BP integration). At graphene Dirac point, n_{Gr} = 0 that requires $D_{top} = D_{bottom}$, indicating $\frac{\mathrm{d}V_{\mathrm{tg}}}{\mathrm{d}V_{\mathrm{bg}}} = -\frac{d_{\mathrm{top}}\varepsilon_{\mathrm{bottom}}}{\varepsilon_{\mathrm{top}}d_{\mathrm{bottom}}}$. Therefore, the gate response of mobile graphene charges can be identified as the slope of charge neutral point (CNP)

marked by the black dashed lines. The slope values of $\rho_{102,98}$ and $\rho_{51,67}$ are identical, indicating an isotropic graphene capacitance C_t as well as the BP top surface carrier density. V_{bg} = 0 V separates the CNP line into two parts, depleted BP at $V_{bg} \geq 0$ V and activated BP at $V_{bg} < 0$ V. At $V_{bg} \geq 0$ V, the finite slope of dV_{tg}/dV_{bg} ($\approx$ -0.013) indicates that D_{bottom} originated from p-doped silicon penetrates BP to tune the graphene carrier density. At $V_{bg} < 0$ V, dV_{tg}/dV_{bg} equals 0, suggesting a complete screening of D_{bottom} by activated BP holes.

With these information in mind, we further unveiled the electrostatic screening of BP top channels by repeating the dual-gated mapping measurements at different temperatures. The sheet resistance $\rho_{102,98}$ as functions of V_{bg} and V_{tg} at T = 1.5 K, 50 K, 80 K, 120 K are shown in Figure S14a-14d, respectively. It is interesting to note that the dV_{tg}/dV_{bg} of Gr CNP line (marked by the black dashed lines) varies with temperature. At low temperature, dV_{tg}/dV_{bg} remains 0 for depleted BP, indicating a complete screening of D_{bottom}. At higher temperature T = 50 K, a slightly bending of CNP line with dV_{tg}/dV_{bg} = -0.007 was observed at $V_{bg} > 30$ V, indicating an incomplete screening of D_{bottom}. With further increasing the temperature, dV_{tg}/dV_{bg} increases to -0.008 (80 K) and -0.013 (120 K). We ascribe this anomalous screening effect to the decreased electrostatic screening capability of BP top channels states at increasing temperature. At depleted BP, penetrated D_{bottom} tunes the Fermi energy of both graphene and BP top channel states. Furthermore, the presence of finite in-gap states in depleted BP influences the resistance of graphene at its charge neutral point, as illustrated in Figure 2d of the main text. The augmentation in the BP substrate charge density increases the graphene's Dirac point resistance. As the gate voltage approaches the threshold one, the charged BP in-gap state amplifies the Coulomb scattering and consequentially enhances the resistance at the graphene's Dirac point.

It is ascribed that the negatively correlated n(T) comes from the reduced density of states in BP top channel at higher temperature. In the pristine Gr on BP region, we observed the electrostatic screening effect owing to the interlayer charge transfer. Moreover, due to the thermally increased BP bandgap, the density of BP top channel

state decreases as increasing temperature, losing the screening capability manifested as the observed increasing dV_{tg}/dV_{bg}.

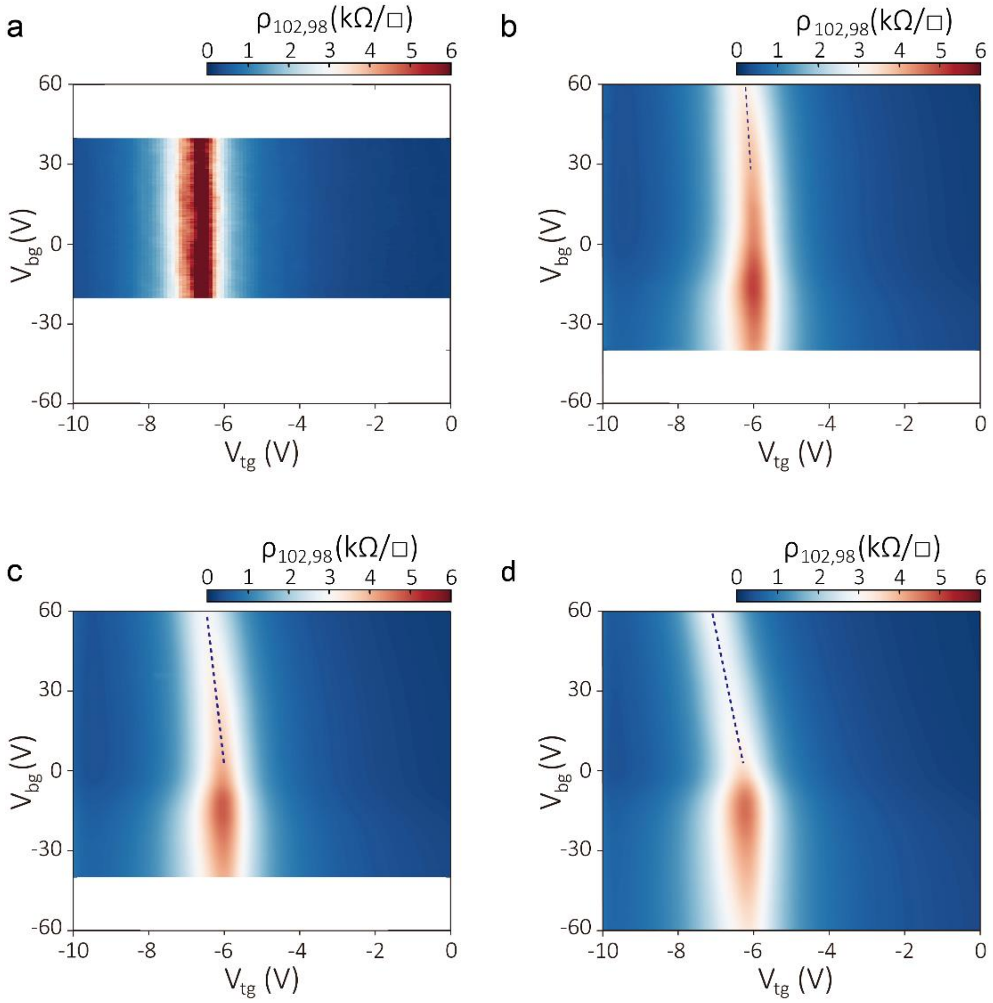

Figure S14 | Electrostatic screening effect in graphene-based transistor with well-aligned Gr on BP. Dual-gated maps of sheet resistance $\rho_{102,98}$ at T = 1.5 K (**a**), 50 K (**b**), 80 K (**c**) and 120 K (**d**). Black dashed lines mark the Gr charge neutral point.

For comparison, we performed the comparative measurements on 2°-1L Gr/BP device (D3, BP thickness ~ 19 nm) where the BP surface strain is ignorable. Figure S15 shows the dual-gated maps of four-terminal resistance at different magnetic fields and temperatures. The slope values of graphene Dirac peak (dV_{tg}/dV_{bg} = -0.064) at depleted BP (yellow dashed lines) is unchanged, indicating that the state number in BP top channel can be ignored. At B = 0 T, the bulk resistance in D3 device shows a step-like feature corresponding to the bipolar nature of BP bottom channel. The absence of BP top channel states allows tuning the BP Fermi energy towards the conduction band.

Furthermore, we observed a pronounced disparity in the carrier mobilities with electron mobility (~ 14,000 cm^2 V^{-1} s^{-1}) substantially lower than the hole mobility (~ 20,000 cm^2 V^{-1} s^{-1}), a phenomenon consistent with observations in Figure S13b. This discrepancy in mobility values can be attributed to the Coulombic interactions between charges in the graphene and holes at the surface of the BP. In this interaction paradigm, electrons within the graphene are attracted to the holes present on the BP surface. This Coulombic attraction tends to localize these electrons, consequently causing a reduction in their mobility, and thus providing an explanation for the observed difference in mobility values.

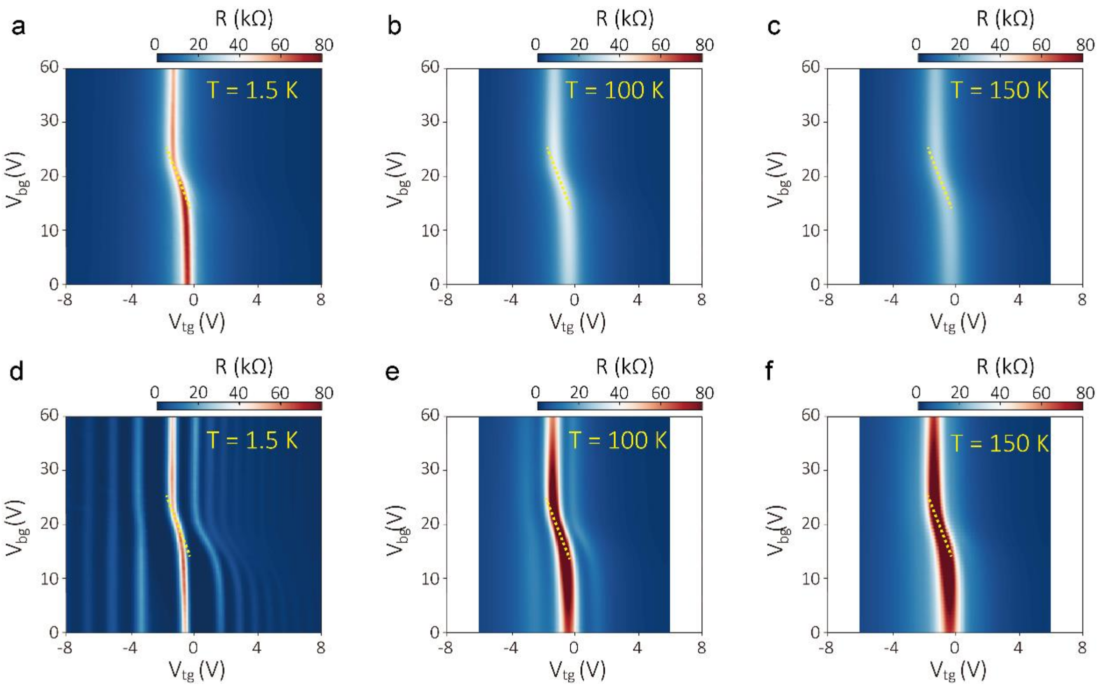

Figure S15 | Screening effect in 2°-Gr/BP device (D3). **a-c**, Dual-gated maps of four-terminal resistance at B = 0 T, T = 1.5 K (**a**), 100 K (**b**), 150 K (**c**). **d-f**, Dual-gated maps of four-terminal resistance at B = 3 T, T = 1.5 K (**d**), 100 K (**e**), 150 K (**f**). Yellow dashed lines mark the CNP of graphene where Gr senses both top and bottom gate fields.

2.6. **Landauer-Büttiker transmissions**

To calibrate the transmission behavior, Landauer-Büttiker formula was adopted as follow

$$I_m = \frac{2e^2}{h} \sum_{n \neq m} (T_{nm} V_m - T_{mn} V_m) \quad \text{(S5)}$$

where m, n denote the electrode labels and T_{mn} refers to the transmission between electrodes m and n that equals the transmission modes multiplying with the transmission probability of each mode. The inter-electrodes transmission can be estimated by injecting the current from different electrodes and measuring the voltages of rest electrodes. Here, all the data was taken at $T = 1.5$ K and $B = 0$ T (current bias is set as 10 nA). $V_{bg} = 50$ V is set to ensure BP depleted.

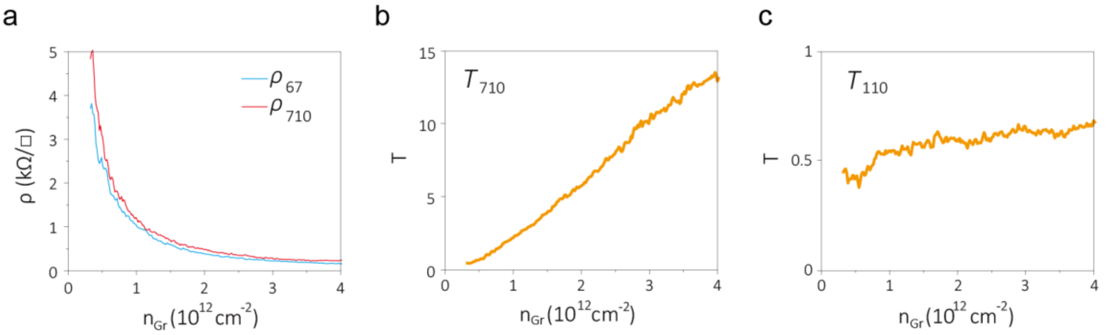

Figure S16 | Landauer-Büttiker transmission in hetero-strain junction. **a**, Sheet resistance of ρ_{67}, ρ_{710} as function of n_{Gr}. **b**, **c**, Transmission matrix elements as function of n_{Gr}, T_{710} (b), T_{110}(c). Measurement was conducted at $B = 0$ T, $T = 1.5$ K, $V_{bg} = 50$ V.

Figure S16a shows the sheet resistances $\rho_{51,67}$ and $\rho_{51,710}$ of the pristine Gr in X-Hallbar. $\rho_{51,710}$ is found to be slightly larger than $\rho_{51,67}$, indicating the reducing effect channel width between electrodes 7 and 10. To find more accurate values of T_{710} and T_{110}, current was injected from different electrodes and measured the voltages at rest electrodes. The transmissions T_{710}, and T_{110} were extracted as shown in Figure S16b-16c. The transmission T_{110} (~ 0.5) is due to the interlayer coupling that generates the nearly flat band with reduced resistivity. In the meantime, T_{710} is one order of magnitude higher than the T_{110}.

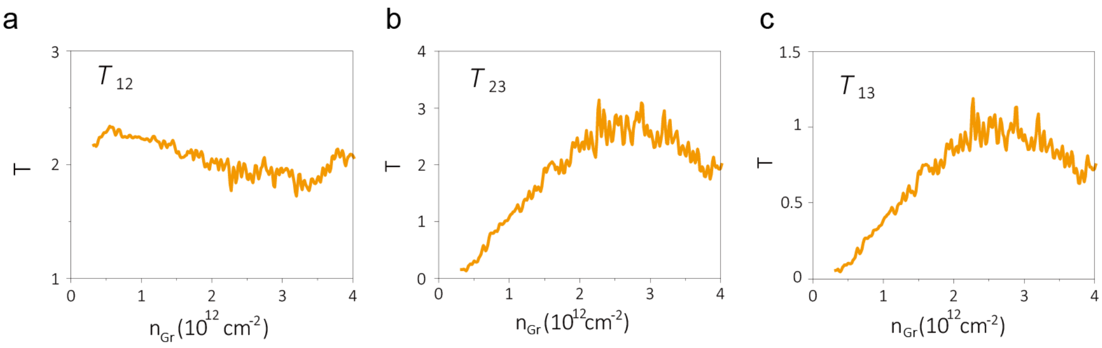

Figure S17 | Landauer-Büttiker transmission of strained graphene on BP. a-c, Transmission matrix elements as function of n_{Gr}, T_{12} (**a**), T_{23} (**b**), T_{31} (**c**). Measurement was conducted at $B = 0$ T, $T = 1.5$ K, $V_{bg} = 50$ V.

The large transmission in pristine Gr allows us to simplify the discussion by viewing electrodes 4, 5, 6, 7 as one electrode (denoted as 7), electrode 8, 9, 10, 11, 12 as one electrode (denoted as 10). According to the formula S5, any non-zero value of T_{ix}, i = 4, 5, …, 12, x = 2, 3, produces the voltage signal at electrodes 2 and 3 when applying current I_{51} or I_{15}. The forbidden transmission across the reflective interface indicates T_{ix} = 0, i = 4, 5, …, 12, x = 2, 3. By applying current I_{21} (between electrodes 2 and 1) and I_{23}, I_{13}, I_{35}, I_{46}, T_{12}, T_{23} and T_{13} were extracted in Figures S17a-17c, respectively. T_{23} and T_{13} reduce at lower graphene carrier density n_{Gr} while T_{12} maintains 2.0±0.3 for varying n_{Gr}. The observed finite transmissions in strained graphene can be attributed to the emergence of edge states following partial strain relaxation, most prominently at the junction of electrodes 1 and 2[S15]. The bulk carrier density is externally modulated by gating, effectively counterbalancing the electrostatic energy. Notably, within the region demarcated by electrodes 1 and 2, there exists a device corner potentially conducive to charge accumulation. This, in turn, reshapes the local electrostatic energy profile. In such a context, the overarching influence of the top-gate voltage on charge conveyance between electrodes 1 and 2 remains subdued, yielding a largely invariant behavior to the carrier density variations.

2.7. **Single wave propagation through reflective interface**

Figure S18 exhibits a rectangular graphene sample (128 nm × 74 nm) with 360000 carbon atoms, which is partly strained along the armchair direction at the low-right corner. The strained pattern was modeled based on STM data. As the initial electron wave function, a Gaussian wave-packet located at the left end of the sample. Then, we investigated the propagation of one-dimensional Gaussian wave-packet within the sample, following the procedure of tight-binding propagation method[S16,S17]. We propagated the wave function by thousands of steps from the left to the right of the sample, and saved the snapshots of the last step. The snapshots were visualized by the wave functions as shown in Figure S18, where the wave-packet diffuses freely and meets the strained boundaries.

For an amplitude (Δz) as low as 50 pm, the wave function propagates freely at pristine graphene but is blocked at the reflective interface in between strained graphene and pristine graphene. The direct junction transmission at armchair direction is significantly diminished whereas the charge depletion area is recharged via zigzag direction. To introduce structural disorders, we randomly added several bump deformations (diameter ~1 nm) with the height of 50~100 pm. Then the depletion area at strained graphene increases due to the enhanced charge reflection (Figure S18b). For the amplitude (Δz) up to 100 pm (Figure S18c), the depletion area is increased with respect to the low-strain circumstance. With random disorders (Figure S18d), we found a complete charge depletion at strained graphene where the charge transmission at zigzag direction is blocked, in consistent with our LB results.

In the aspect of the strain-free region, graphene sustains its Dirac-type carriers and exhibits a mobility up ~ 20,000 $cm^2V^{-1}s^{-1}$. By local thermal-annealing of graphene and BP, the in-plane reflective interface at the joint point of two distinct regions is capable to modify the direction of charge flow, leading to remarkable voltage regulations.

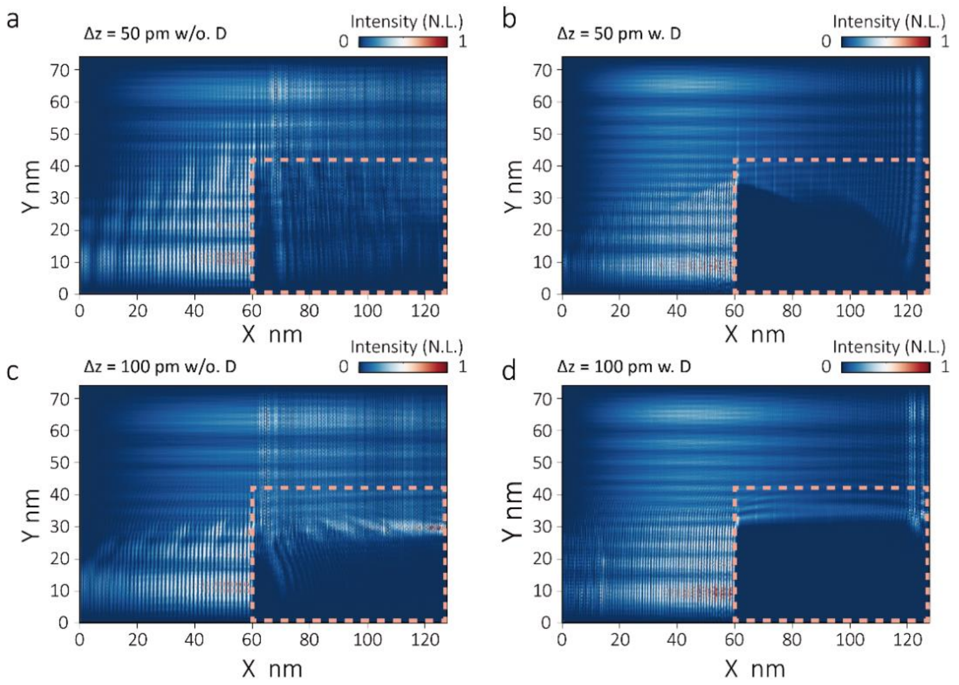

Figure S18 | Theoretical simulation of single particle wave propagation at graphene with reflective interface. a, **b**, Single particle wave propagation across the strained graphene where the out-of-plane deformation amplitude (Δz) is set as 50 pm, without structural disorders (**a**) and with structural disorders (**b**). **c**, **d**, Single particle wave propagation across the strained graphene where the out-of-plane deformation amplitude is set as 100 pm, without structural disorders (**c**) and with structural disorders (**d**). The intensity refers to the normalized probability amplitude. Orange dashed rectangles mark the strained graphene area while the rest graphene remains flat.

2.8. **Gate tunable nonlocal results**

The nonlocal measurement was performed by injecting current from electrodes 10 to 2 (I_{102}) and measuring $R_{102,73} = V_{102,73}/I_{102}$, $R_{102,64} = V_{102,64}/I_{102}$. Figure S18a-18f exhibit the $R_{102,73}$ and $R_{102,64}$ at T = 50 K, 80 K and 120 K. From these data, three features appear. Firstly, at V_{bg} < 5 V, a significant nonlocal signal $R_{102,73}$ raises up to 10^4 Ω whereas $R_{102,64}$ maintains zero for varying V_{tg}. This is because the inter-junction transmission $T_{ix} = 0$ (i = 4, 5, …, 12, x = 2, 3) that prohibits current passing through electrodes 4, 5, 6, 7. From the equation S5, $V_{102,3} = \frac{T_{31}}{T_{31}+T_{32}} V_{102,1} \approx 0$ is significantly smaller than $V_{102,7} = \frac{T_{71}V_{102,1}+T_{710}V_{102,10}}{T_{17}+T_{710}} = V_{102,4} = V_{102,6}$. In this way, the nonlocal voltage $V_{102,64}$ becomes zero at depleted BP.

Secondly, the nonlocal resistance $R_{102,73}$ reaches its maximum at the graphene Dirac point. Note that the slope (dV_{tg}/V_{bg}) of $R_{102,73}$ in this region varying with temperature originates from the electrostatic screening effect of BP top channel, in good agreement with the local transport results (Figure S14).

Thirdly, the nonlocal signal for both $R_{102,73}$ and $R_{102,64}$ depends on the activation of BP. The activated BP switches T_{ix} (i = 4, 5, …, 12, x = 2, 3) from zero to a finite value and decreases $R_{102,73}$. Moreover, the nonlocal signal $R_{102,64}$ also switches from zero to a finite value because of BP bridging current.

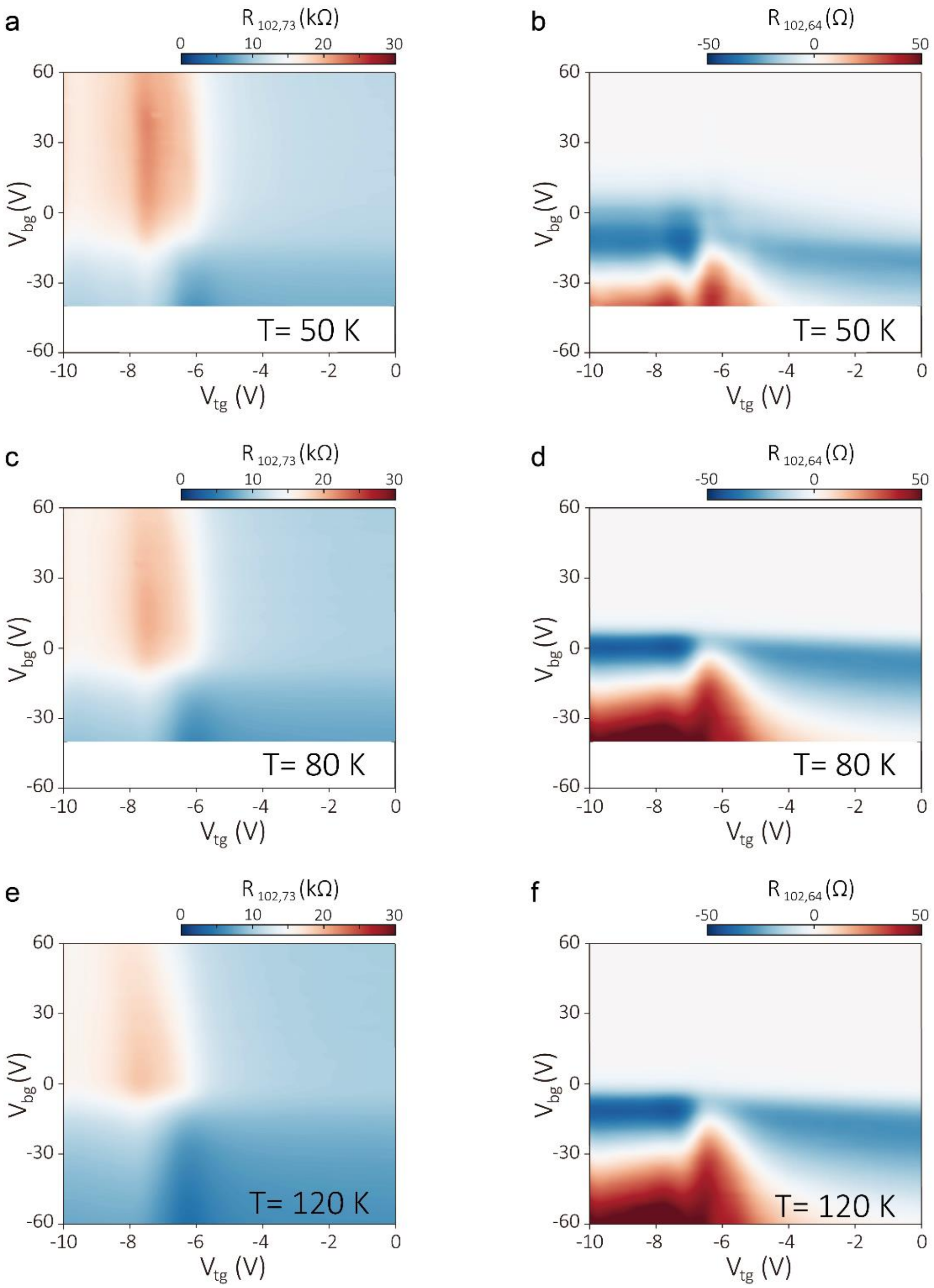

Figure S19 | Nonlocal results of graphene-based transistor. **a**, **b**, Dual-gated maps of nonlocal resistance $R_{102,73}$ (**a**) and $R_{102,64}$ (**b**) at T = 50 K. **c**, **d**, Dual-gated maps of nonlocal $R_{102,73}$ (**c**) and $R_{102,64}$ (**d**) at T = 80 K. **e**, **f**, Dual-gated maps of nonlocal results $R_{102,73}$ (**e**) and $R_{102,64}$ (**f**) at T = 120 K.

2.9. **Magneto-response of inter-junction resistance**

To investigate the charge transport at the reflective interface, we applied perpendicular magnetic fields (B) in the quantum Hall regime. Figure S20a shows the inter-junction resistance $R_{\text{inter}}(R_{51,43})$ as function of V_{tg} and B at T = 1.5 K. It is found that R_{inter} is strongly asymmetric with respect to magnetic field especially at the QH states, in contradicted to the conventional edge modes in the QH theory. Moreover, we observed the addition magneto-oscillations manifested as horizontal streaks that are independent with n_{Gr}.

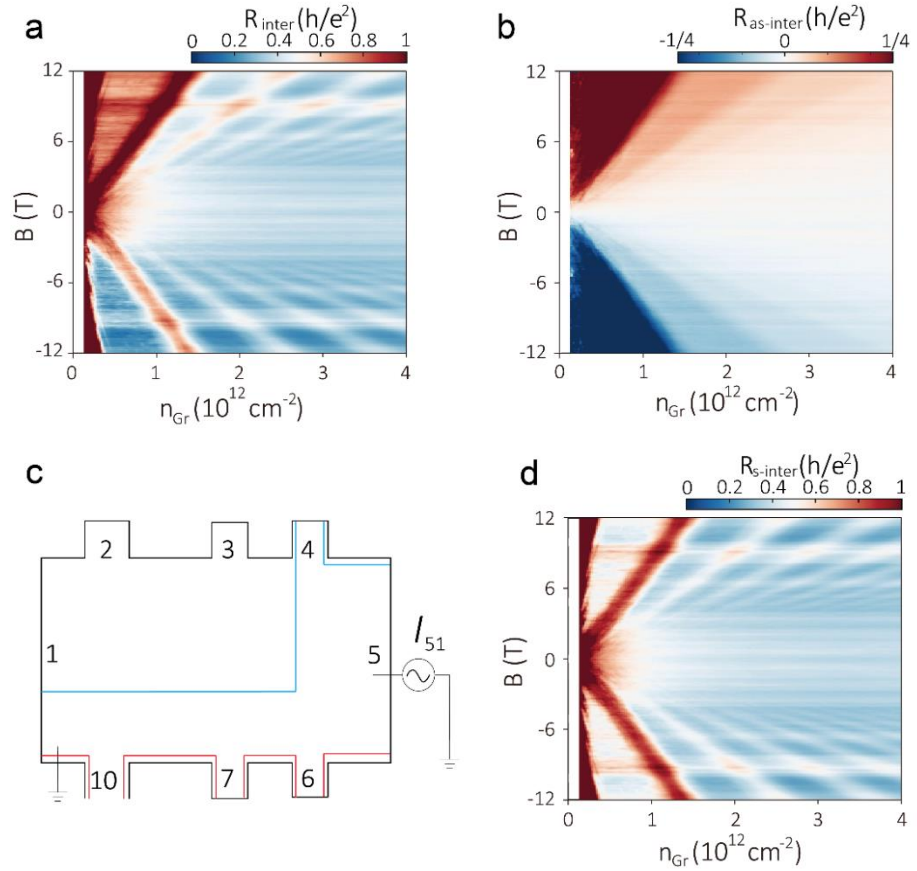

Figure S20 | Magneto-response of inter-junction resistance. **a**, Inter-junction resistance R_{inter} ($R_{51,43}$) as function of magnetic field and n_{Gr} at T = 1.5 K, V_{bg} = 60 V. **b**, Asymmetric component of R_{inter} denoted as $R_{\text{as-inter}}$ as function of magnetic field and n_{Gr}. **c**, Schematic of quantum Hall edge channels. **d**, Symmetric component of R_{inter} denoted as $R_{\text{s-inter}}$ as function of B and n_{Gr}.

To explain these observations, we extracted the B-asymmetric component $R_{\text{as-inter}}$ according to $R_{\text{as-inter}} = 0.5(R_{\text{inter}}(+B) - R_{\text{inter}}(-B))$. Figure S20b shows the extracted $R_{\text{as-inter}}$ in unit of h/e^2. The asymmetric component $R_{\text{as-inter}}$ exhibits a resistance plateau behavior at each QH states (where the plateau resistance equals to $h/2ve^2$, v is the

corresponding filling factor). The magneto transport could be divided into two parts, quantized edge transport at pristine graphene and resistive transport (finite transmission T_{110}). The schematic of edge channels at pristine graphene is shown in Figure S20c in light of complete charge reflection at the strain junction. According to the QH theory, the backscattering is forbidden at the edge channels. As a result, $V_{51,4} = V_{51,1} = 0$ (blue channels) and $V_{51,10} = V_{51,7} = V_{51,6} = V_{51,5} = hI_{51}/ve^2$ (red channels) at QH states (filling factor v). After reversing the direction of magnetic field, $V_{51,2} = V_{51,3} = 0$ remains. Therefore, $V_{51,4} = V_{51,5} = hI_{51}/ve^2$, $V_{51,10} = V_{51,7} = V_{51,6} = V_{51,1} = 0$, leading to Hall-like $V_{51,43}$ extracted via $(V_{51,43}(+B) - V_{51,43}(-B))/2I_{51} = h/2ve^2$, in consistent with our observations.

Except for the asymmetric behavior, the symmetric component $R_{\text{s-inter}} = 0.5[R_{\text{inter}}(+B) + R_{\text{inter}}(-B)]$ inherits additional oscillation peaks as shown in Figure S20d. These oscillations in $R_{\text{s-inter}}$ suggests the fractal states in Gr/BP superlattice. At these fractal states, sample resistance reaches its local minimum. To highlight these fractal states that is independent with n_{Gr}, we plotted the inter-junction resistance $R_{51,43} = V_{51,43}/I_{51}$ as function of B in Figure S21. Oscillation periodicity $\Delta B = 0.5\pm0.3$ T is observed, indicating the existence of superlattice and related magnetic fractal states [S18, S19].

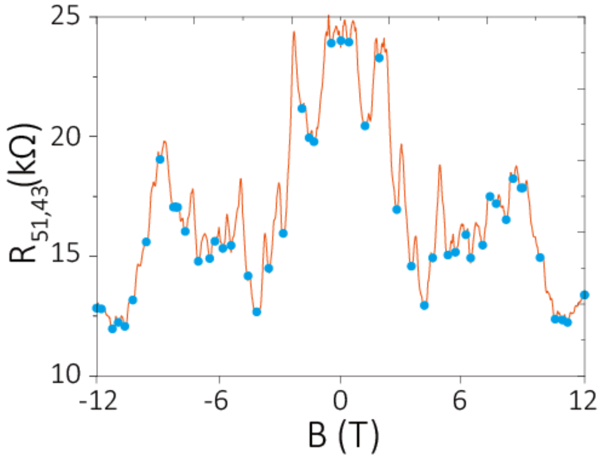

Figure S21 | Magneto response of inter-junction resistance $R_{51,43}$ at T= 1.5 K, V_{tg} = 0 V and V_{bg} = 60 V. Blue dots mark the local resistance minimum at the fractal states.

2.10. Differential conductance in multi-terminal graphene-based transistor

The energy-dependent differential conductance characterizes the charge transport behavior at varying electrostatic potentials. Figure S22b-22c show the differential conductance as function of DC bias between electrodes 2 and 3 (b), 6 and 7 (c), respectively. At the strained graphene, the differential conductance shows a local maximum at zero DC bias, in contradiction to the conventional graphene behavior (Figure S22c). The differential conductance between electrodes 2 and 3 is mainly from graphene edge channels after partial strain released. According to the calculated band structure of the strained graphene, the nearly flat band at zero energy gives the largest density of states. Hence, the observed differential conductance peak at $|V_{\mathrm{bias}}| < 1.5$ mV is mainly from charge hopping among localization centers that highly depends on the density of states.

Figure S22d-22f show the inter-junction differential conductance as function of DC bias between electrodes 4 and 3 (d), 7 and 3 (e), 2 and 10 (f). In comparison with $\mathrm{d}I_{23}/\mathrm{d}V$ and $\mathrm{d}I_{67}/\mathrm{d}V$, all curves show reduced conductance, indicating a decreasing number of conducting channels, especially in the strained graphene. In addition, $\mathrm{d}I_{34}/\mathrm{d}V$, $\mathrm{d}I_{37}/\mathrm{d}V$ and $\mathrm{d}I_{210}/\mathrm{d}V$ show the turning points at $|V_{\mathrm{bias}}| = 4$ mV that is absent for pristine Gr (Figure S22c, $\mathrm{d}I_{67}/\mathrm{d}V$), suggesting the saturation of conducting channel in the strained Gr. It is also worthy to highlight the higher differential conductance at negative DC bias than positive DC bias. The loss of reciprocity at $|V_{\mathrm{bias}}| > 4$ mV is due to the presence of accumulated charges at the edge (in between electrodes 1 and 2) that alter the in-plane electric field. These edge states dominate the charge transport between electrodes 1 and 2 manifested as gate invariable T_{12} (Figure S17a), and break the DC bias reciprocity.

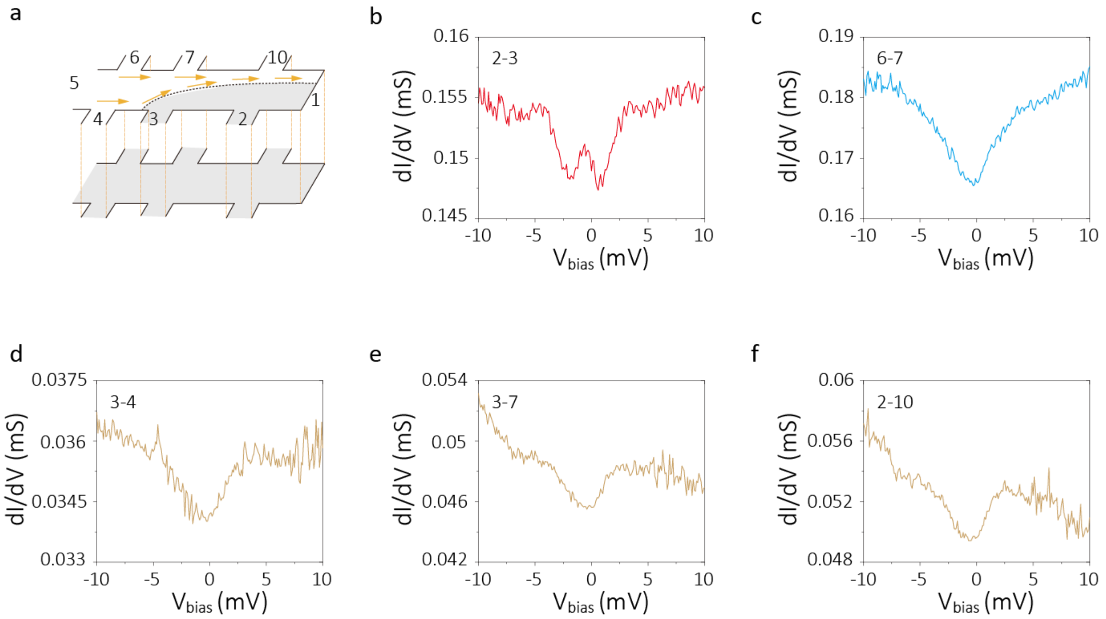

Figure S22 | Differential conductance of multi-terminal graphene-based transistor. **a**, Schematic of multi-terminal configuration. **b**, **c**, Differential conductance of the strained Gr (b) and pristine Gr (c) as function of DC voltage bias. **d-f**, Inter-junction differential conductance as function of DC voltage bias between electrodes 3 and 4 (d), 3 and 7 (e), 2 and 10 (f).

3. Parallel propagation

3.1. Evidences of parallel propagation

The activation of BP is the core to achieve the field effect of graphene-based transistor. Leveraging the advantages of a bottom gate, we can facilely manipulate the Fermi energy of BP. For instance, a positive voltage applied to the bottom gate leads to the depletion of BP, as shown in Figure S23a. At this state, the electrostatic gating penetrates through insulating bulk BP and successfully tunes the charge distribution within the upper BP channel. In contrast, the imposition of a negative voltage to the bottom gate (refer to Figure S23b) induces charge accumulation within bottom BP layer. These accumulated charges facilitate parallel conduction and effectively screen the electric field from the bottom gate, therefore, contributing to the distinctive behavior of this system.

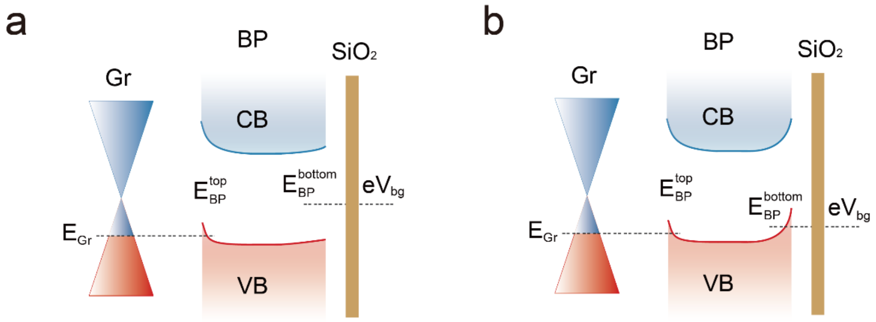

Figure S23 | Schematic of graphene/BP band alignments with bottom BP channel deactivated (a) and activated (b), respectively.

After activated, BP and graphene form a parallel propagation mode. For parallel propagation, the longitudinal resistivity ρ_{xx} and Hall resistivity ρ_{xy} can be expressed [S20]

$$\rho_{xx} = \frac{\rho_B(1+\beta_T^2)(1+\beta_B^2)[(1+\beta_T^2)+\alpha(1+\beta_B^2)]}{[(1+\beta_T^2)+\alpha(1+\beta_B^2)]^2+[\beta_B(1+\beta_T^2)+\alpha\beta_T(1+\beta_B^2)]^2} \quad (S6)$$

$$\rho_{xy} = \frac{\rho_B(1+\beta_T^2)(1+\beta_B^2)[\beta_B(1+\beta_T^2)+\alpha\beta_T(1+\beta_B^2)]}{[(1+\beta_T^2)+\alpha(1+\beta_B^2)]^2+[\beta_B(1+\beta_T^2)+\alpha\beta_T(1+\beta_B^2)]^2} \quad (S7)$$

where $\rho_{T,B}$ is resistivity of each layer, $\beta_{T(B)} = \mu_{T(B)}B$ and $\alpha = \rho_B/\rho_T$. At $B = 0$ T, the formula S6 can be simplified as $\rho_{xx} = \rho_T\rho_B/(\rho_T + \rho_B)$, $\rho_{xy} = 0$. Accordingly, $R_{Bij} = (e\mu_{BP}n_{BP})^{-1}$ decreases with decreasing V_{bg} so as the total resistance R_{Pij}. To disclose the relationship, we performed measurement on $V_{51,67}$ at pristine graphene as functions of V_{tg} and V_{bg} (Figure S24a). Except for the electrostatic screening from activated BP

manifested as $dV_{tg}/dV_{bg} = 0$ (orange dashed line), $V_{51,67}$ decreases with decreasing V_{bg}. The parallel propagation coefficient $\eta = V_{51,73}/V_{51,73}$ (V_{tg} = 0 V) is defined based on transverse signal in between electrodes 7 and 3 as shown in Figure S24b.

Furthermore, in the quantum Hall regime, equation S6 is not applicable due to the vanishing diagonal resistivity as well as diagonal conductivity of graphene. Figure S24c shows the $V_{51,67}$ as functions of V_{tg} and V_{bg} at B = 12 T. The $V_{51,67}$ remains 0 at depleted BP while increases at activated BP. Figure S24d shows the increasing $V_{51,67}$ at quantum Hall states extracted at V_{tg} = -4 V, mainly due to the diffusive transport at BP.

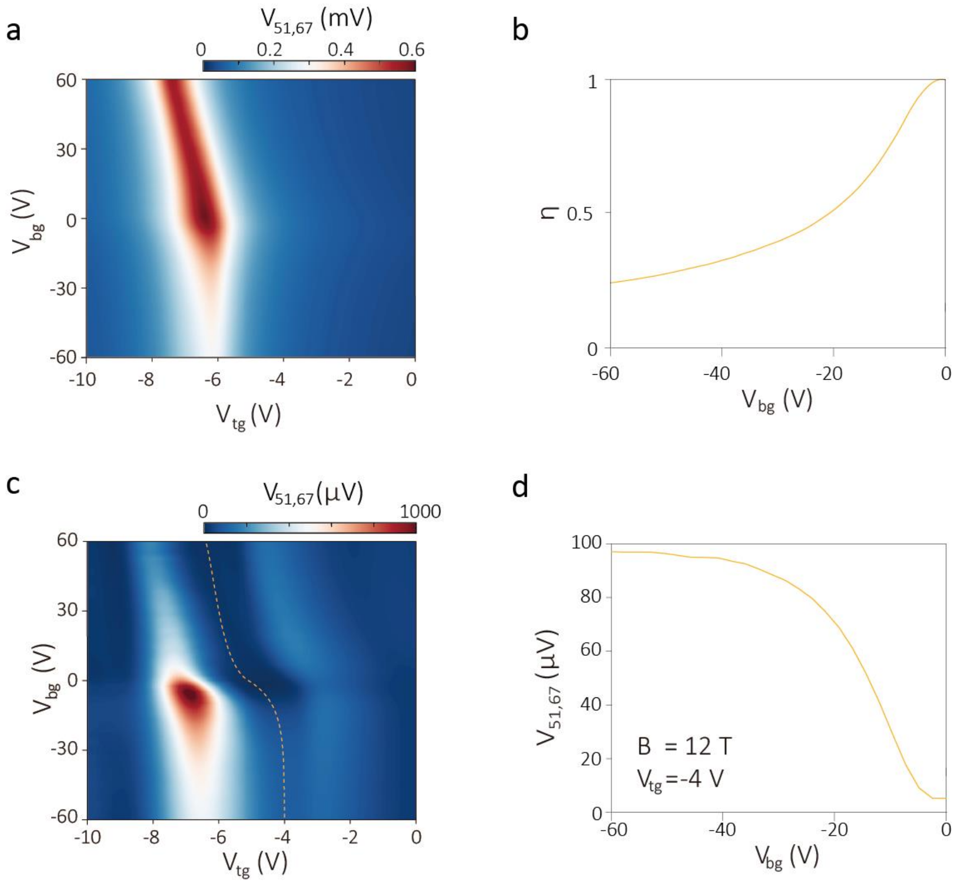

Figure S24 | Parallel propagation in graphene-based transistor. a, Dual-gated maps of $V_{51,67}$ at I_{bias} = 100 nA, T = 120 K. **b**, Parallel propagation Coefficient η as function of V_{bg}. **c**, Dual-gate mapping of $V_{51,67}$ in the quantum Hall regime at I_{bias} = 100 nA, T = 120 K, B = 12 T. Orange dashed line mark the position of v = 2. **d**, Non-zero voltage $V_{51,67}$ as function of V_{bg} at V_{tg} = -4 V (v = 2).

3.2. Theoretical simulation of longitudinal $V_{51,32}$

When BP is depleted ($V_{bg} \geq 0$), no current flows in strained graphene gives $V_{51,1} = V_{51,2} = V_{51,3} = 0$ and then $V_{51,32} = 0$. The voltage difference between the electrodes 3 and 2 can be expressed as

$$V_{51,32} = V_{51,102} + V_{51,710} + V_{51,37} \tag{S8}$$

When current is absent, we obtain $V_{51,102}(0) = V_{51,10}$, $V_{51,37}(0) = -V_{51,7}$ and $V_{51,32} = V_{51,10} + (V_{51,7} - V_{51,10}) + (-V_{51,7}) = 0$. When BP bottom channel is on ($V_{bg} < 0$), the voltage differences between electrodes 3 and 7 ($V_{51,37}$), and 10 and 2 ($V_{51,102}$) drive a Hall-like transverse current that results in a screening potential. It in turn reduces their voltage potential differences ($V_{51,102}$ and $V_{51,37}$). We assume the parallel propagation is isotropic between pristine graphene and BP, i.e. $V_{51,102} = \eta V_{51,102}(0) = \eta V_{51,10}$ and $V_{51,37} = \eta V_{51,37}(0) = -\eta V_{51,7}$. We then have

$$V_{51,32} = \eta V_{51,10} + (V_{51,7} - V_{51,10}) + (-\eta V_{51,7}) = (1-\eta) V_{51,710} \tag{S9}$$

$\eta = 1$ corresponds to the depleted BP, which gives $V_{51,32} = 0$. With the hole accumulation in BP, η decreases and the screening effect becomes more distinct. Based on the experimental $V_{51,37}$, Figure S23b shows the parallel propagation coefficient $\eta = V_{51,73}(0)/V_{51,73}$ as function of V_{bg}. As can be seen, activated BP gives a nonzero voltage difference between the electrodes 3 and 2 ($V_{51,32}$) that is proportional to $V_{51,710}$.

The difference between electrodes 7 and 10 can be expressed as $V_{51,710} = R^G \cdot I^T$, with $R^G = \frac{\alpha}{(V_{tg}-V_{DC})^2+\lambda^2}$, where V_{DC} is the voltage of Dirac point, λ is its half width and α is a parameter determining its amplitude. I^T is the current in graphene. Therefore, based on equation S9, Figure 2e shows the simulated $V_{51,32}$ as functions of top and bottom gate voltages ($V_{DC} = -6.2$ and $\lambda = 1$), which agrees well with our experimental results (Figure 2b).

3.3. Theoretical calculation of transverse $V_{51,73}$

When $V_{bg} \geq 0$ V, there exist no current flow in strained graphene and we then have $V_{51,3} = 0$. In this case, $V_{51,73}$ is then totally determined by $V_{51,7} = V_{51,71}$. The voltage at

electrode 7 is then $V_{51,7} = R^G_{51,71} \cdot I^t$, where $R^G_{51,71}$ is the graphene resistence between the electrodes 1 and 7. We assume that the dole-gate (V_{tg} and V_{bg}) dependence of the resistance has a form of $R^G_{51,71} = \frac{\alpha(1-\beta V_{bg})}{(V_{tg}+\gamma V_{bg}-V_{DC})^2+\lambda^2}$, where β accounts for additional conducting channel from bottom BP at $V_{bg} < 0$, and γ is the efficiency ratio of the top and bottom gates.

When BP bottom channel is activated ($V_{bg} < 0$V), the Hall-like current starts to average the voltage difference between electrodes 7 and 3, i.e. $V_{51,73} = \eta V_{51,73}(0)$. Figure 2f shows the simulated $V_{51,73}$ as functions of top and bottom gate voltages ($\beta = 0.003$ and $\lambda = 0.02$), which agrees well with our experimental results (Figure 2d).

3.4. Simulating the negative $V_{51,32}$ under magnetic fields

At $V_{bg} < 0$V, the smearing of the Hall-like voltage originates from the transverse bridging current in BP bottom channel that is proportional to $\eta V_{51,710}$. In the presence of a perpendicular magnetic field, this current creates a Hall component between electrodes 3 and 2. Because the Hall component is proportional to the current and inversely proportional to carrier density, we derived a formula $V^H_{51,32} = \eta V_{51,710}(B)\frac{\zeta(B)}{V_{bg}+\lambda}$, where $\zeta(B)$ is a fitting parameter and λ is introduced to eliminate the divergence at $V_{bg} = 0$ V. Since $V_{51,710}$ and $V_{51,67}$ are both measured at pristine graphene, one may adopt experimental results of $V_{51,67}(B)$ to simulate $V_{51,710}(B)$ up to a coefficient ϑ relating to their length difference. The total voltage difference between electrodes 3 and 2 is rewritten as

$$\mathrm{V}_{51,32}(B) = \vartheta\left[(1-\eta)\mathrm{V}_{51,67}(B) + V^H_{51,32}\right] \quad \text{(S10)}$$

Because the sign of $V^H_{51,32}$ depends on the direction of magnetic field, this Hall component would compete with the first term when they have opposite signs.

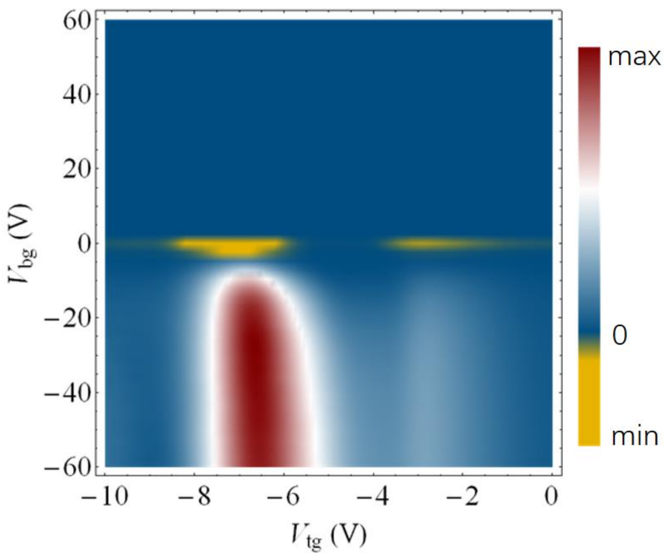

Figure S25| Simulated $V_{51,32}(B)$ as functions of top and bottom gate voltages in the presence of a magnetic field B. $\zeta(B) = 0.2$ and $\lambda = -1$ are used.

4. Temperature-dependence resistance in pristine graphene

Figure S26a-26c show the room-temperature mappings of $R_{51,67}$ as functions of dual-gates at B = -12 T, 12 T and 0 T, respectively. Note that, $R_{51,67}$ increases with decreasing V_{tg} and approaches the CNP of graphene. However, the variation of $R_{51,67}$ with respect to V_{bg} is due to the current redistribution controlled by activating BP bottom channel. The dual-gate dependence maintains for three magnetic fields. Moreover, the positive magneto resistance at (V_{tg}, V_{bg}) = (0 V, 50 V) is shown in Figure S26d. Overall, the magneto-resistance of $R_{51,67}$ is symmetric with respect to the magnetic field, suggesting that the transport of pristine graphene remains classical.

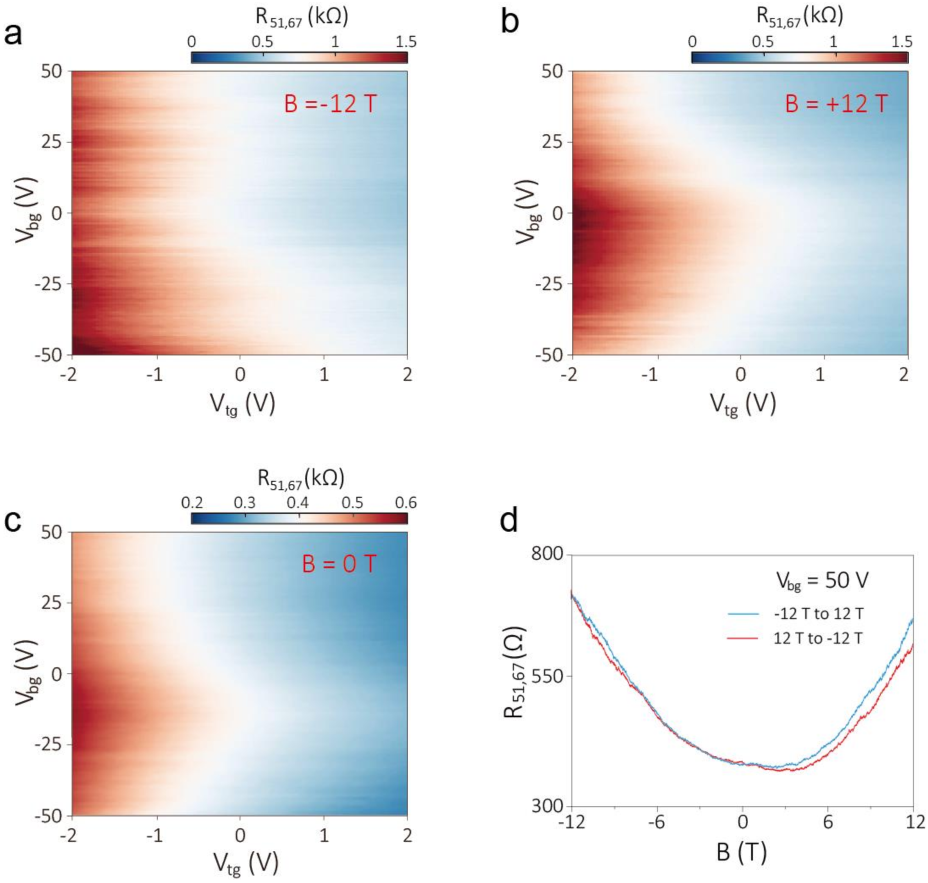

Figure S26 | Temperature-dependent resistance of pristine graphene on BP. a-c, Dual-gate mapping of resistance $R_{51,67}$ at I_{bias} = 100 nA, T = 300 K, B = -12 T (**a**), B = 12 T (**b**), B = 0 T (**c**). **d**, Magneto-resistance of $R_{51,67}$ at V_{bg} = 50 V, V_{tg} = 0 V.

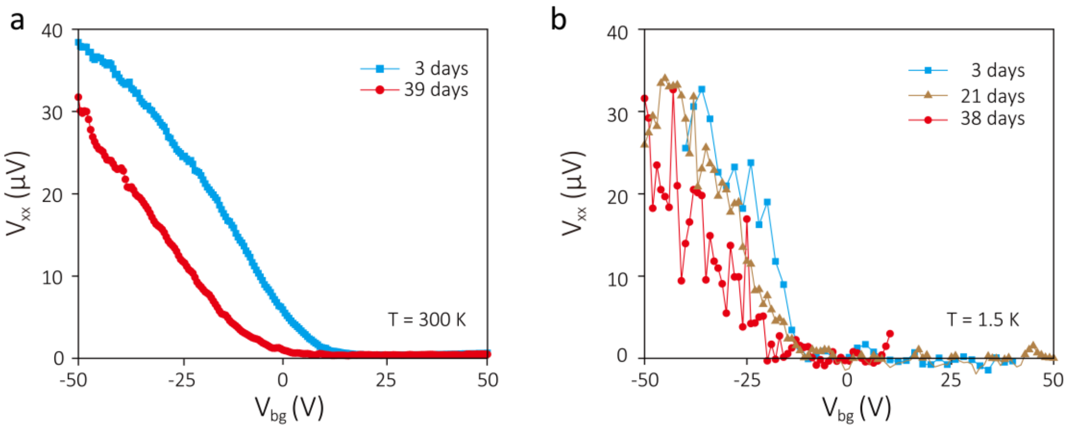

Figure S27 | Stability test of the graphene transistor at 300 K (a) and 1.5 K (b).

We performed the stability test of our graphene transistor for over one month. The room temperature and low temperature switching curves are well-maintained after 39 days as shown in Figs. S27a and b.

5. Performance summary of assorted field effect transistors

Table S1: on/off ratios and charge mobilities in various field effect transistors

Material	on/off ratio	Mobility ($cm^2 V^{-1} s^{-1}$)	Reference
Silicon on insulator (SOI)	10^5	1000	S21
AlGaN/GaN	10^2	1500	S22
$PdSe_2$	10^3	216	S23
$SnSe_2$	10^5	85	S24
WS_2	10^7	50	S25
	10^7	214	S26
WSe_2	10^7	140	S27
	10^5	142	S28
MoS_2	10^8	17	S29
	10^6	100	S30
	2.5×10^6	34	S31
Rippled MoS_2	10^6	900	S32
$MoTe_2$	10^3-10^5	50-130	S33
ReS_2	10^4	7.6	S34
$ZrSe_2$	10^5	0.1	S35
$HfSe_2$	10^6	0.3	
$PtSe_2$	10^3	210	S36
Black Phosphorus	10^5 - 10^8	100-2000	S37
Black Arsenic	10^5	51-4000	S38
Black Arsenic Phosphorus	2-20	79-83	S39
Silicene	10	100	S40
Tellurene	10^6	700	S41
Graphene	2-20	10^4	S42
Graphene Nanoribbon (GNR)	20	880	S43
InSe	10^5 - 10^7	1000	S44, S45
SnSe	2×10^7	5.8	S46
GeAs	10^5	100	S47
Graphene/MoS_2	10^8	100	S48
Organic FET	4×10^5	0.02	S49
ZnO thin film	1.65×10^5	100	S50
Graphene/Pentacene	10^4	220	S51
Electrolyte-Gated Transistors	10^5	1.8	S52
	10^4 - 10^7	10-30	
Polymer semiconductors	10^6	2	S53
Amorphous Zinc Tin Oxide	10^7	50	S54
Amorphous $InGaZnO_4$	10^8	12	S55

6. Reference

S1 Liao, A. D. *et al.* Thermally limited current carrying ability of graphene nanoribbons. *Phys. Rev. Lett.* **106**, 256801 (2011).

S2 Grosse, K. L. *et al.* Nanoscale Joule heating, Peltier cooling and current crowding at graphene-metal contacts. *Nat. Nanotechnol.* **6**, 287-290 (2011).

S3 Huang, S. *et al.* From anomalous to normal: temperature dependence of the band gap in two-dimensional black phosphorus. *Phys. Rev. Lett.* **125**, 156802 (2020).

S4 Balandin, A. A. *et al.* Superior thermal conductivity of single-layer graphene. *Nano Lett.* **8**, 902-907 (2008).

S5 Neumann, C. *et al.* Raman spectroscopy as probe of nanometre-scale strain variations in graphene. *Nat. Commun.* **6**, 8429 (2015).

S6 Levy, N. *et al.* Strain-induced pseudo-magnetic fields greater than 300 tesla in graphene nanobubbles. *Science* **329**, 544-547 (2010).

S7 Tahir, M. *et al.* Emergent flat band lattices in spatially periodic magnetic fields. *Phys. Rev. B* **102**, 035425 (2020).

S8 Liu, Y. *et al.* Tailoring sample-wide pseudo-magnetic fields on a graphene-black phosphorus heterostructure. *Nat. Nanotechnol.***13**, 828-834 (2018).

S9 Li, L. *et al.* Black phosphorus field-effect transistors. *Nat. Nanotechnol.* **9**, 372-377 (2014).

S10 Li, L. *et al.* Quantum Hall effect in black phosphorus two-dimensional electron system. *Nat. Nanotechnol.* **11**, 593-597 (2016).

S11 Saito, Y. *et al.* Gate-tuned thermoelectric power in black phosphorus. *Nano Lett.* **16**, 4819-4824 (2016).

S12 Sheng, D. N. *et al.* Quantum Hall effect in graphene: Disorder effect and phase diagram. *Phys. Rev. B* **73**, 233406 (2006).

S13 Moser, J. *et al.* Magnetotransport in disordered graphene exposed to ozone: From weak to strong localization. *Phys. Rev. B* **81**, 205445 (2010).

S14 Chau, T. K. *et al.* Detection of hidden localized states by the quantum Hall effect in graphene. *Curr. Appl. Phys.* **23**, 26-29 (2021).

S15 Aharon-Steinberg, A. *et al.* Long-range nontopological edge currents in charge-neutral graphene. *Nature* **593**, 528-534 (2021).

S16 Hams, A., & De Raedt, H. Fast algorithm for finding the eigenvalue distribution of very large matrices. *Phys. Rev. E*, **62**, 4365 (2000).

S17 Li, Y. *et al.* TBPLaS: A tight-binding package for large-scale simulation. *Comput. Phys. Commun.* **285**, 108632 (2023).

S18 Krishna Kumar, R. *et al.* High-temperature quantum oscillations caused by recurring Bloch states in graphene superlattices. *Science* **357**, 181-184 (2017).

S19 Krishna Kumar, R. *et al.* High-order fractal states in graphene superlattices. *Proc. Natl. Acad. Sci. U. S. A.* **115**, 5135-5139 (2018).

S20 Harris, J. J. Simplified assessment of parallel conduction in modulation-doped heterostructures. *Meas. Sci. Technol.* **2**, 1201 (1991).

S21 International Roadmap for Devices and Systems 2022: Executive Summary. *IEEE* (2022).

S22 Selvaraj, S. L. *et al.* 1.4-kV breakdown voltage for AlGaN/GaN high-electron-mobility transistors on silicon substrate. *IEEE* **33**, 1375-1377 (2012).
S23 Chow, W. L. *et al.* High mobility 2D palladium diselenide field-effect transistors with tunable ambipolar characteristics. *Adv. Mater.* **29**, 1602969 (2017).
S24 Guo, C. *et al*. Field-effect transistors of high-mobility few-layer SnSe2. *Appl. Phys. Lett.* **109**, 203104 (2016).
S25 Aji, A. S. *et al*. High mobility WS2 transistors realized by multilayer graphene electrodes and application to high responsivity flexible photodetectors. *Adv. Funct. Mater.* **27**, 1703448 (2017).
S26 Iqbal, M. W. *et al.* High-mobility and air-stable single-layer WS2 field-effect transistors sandwiched between chemical vapor deposition-grown hexagonal BN films. *Sci. Rep.* **5**, 10699 (2015).
S27 Movva, H. C. *et al.* High-mobility holes in dual-gated WSe2 field-effect transistors. *ACS Nano* **9**, 10402-10410 (2015).
S28 Liu, W. *et al.* Role of metal contacts in designing high-performance monolayer n-type WSe2 field effect transistors. *Nano Lett.* **13**, 1983-1990 (2013).
S29 Wu, W. *et al.* High mobility and high on/off ratio field-effect transistors based on chemical vapor deposited single-crystal MoS2 grains. *Appl. Phys. Lett.* **102**, 142106 (2013).
S30 Kim, S. *et al.* High-mobility and low-power thin-film transistors based on multilayer MoS2 crystals. *Nat. Commun.* **3**, 1011 (2012).
S31 McClellan, C. J., Yalon, E., Smithe, K. K., Suryavanshi, S. V. & Pop, E. *75th annual device research conference (DRC). IEEE* 1-2 (2017).
S32 Ng, H. K. *et al.* Improving carrier mobility in two-dimensional semiconductors with rippled materials. *Nat. Electron*. **5**, 489-496 (2022).
S33 Zhang, Q. *et al.* Simultaneous synthesis and integration of two-dimensional electronic components. *Nat. Electron*. **2**, 164-170 (2019).
S34 Shim, J. *et al.* High-performance 2D rhenium disulfide (ReS2) transistors and photodetectors by oxygen plasma treatment. *Adv. Mater.* **28**, 6985-6992 (2016).
S35 Mleczko, M. J. *et al.* HfSe2 and ZrSe2: Two-dimensional semiconductors with native high-κ oxides. *Sci. Adv.* **3**, e1700481 (2017).
S36 Zhao, Y. *et al.* High-electron-mobility and air-stable 2D layered PtSe2 FETs. *Adv. Mater.* **29**, 1604230 (2017).
S37 Hong, Y. K. *et al.* Recent progress in high-mobility thin-film transistors based on multilayer 2D materials. *J. Phys. D: Appl. Phys.* **50**, 164001 (2017).
S38 Zhong, M. *et al.* Thickness-dependent carrier transport characteristics of a new 2D elemental semiconductor: black arsenic. *Adv. Funct. Mater.* **28**, 1802581 (2018).
S39 Yuan, S. *et al.* Air-stable room-temperature mid-infrared photodetectors based on hBN/black arsenic phosphorus/hBN heterostructures. *Nano Lett.* **18**, 3172-3179 (2018).
S40 Tao, L. *et al.* Silicene field-effect transistors operating at room temperature. *Nat. Nanotechnol.* **10**, 227-231 (2015).
S41 Wang, Y. *et al.* Field-effect transistors made from solution-grown two-dimensional tellurene. *Nat. Electron*. **1**, 228-236 (2018).
S42 Schwierz, F. J. N. n. Graphene transistors. *Nat. Nanotechnol.* **5**, 487-496 (2010).

S43 Liao, L. *et al.* Top-gated graphene nanoribbon transistors with ultrathin high-k dielectrics. *Nano Lett.* **10**, 1917-1921 (2010).

S44 Tsai, T.-H. *et al.* High-Mobility InSe transistors: the nature of charge transport. *Appl. Mater. Inter.* **11**, 35969-35976 (2019).

S45 Huang, Y.-T. *et al.* High-performance InSe transistors with ohmic contact enabled by nonrectifying barrier-type indium electrodes. *Appl. Mater. Inter.* **10**, 33450-33456 (2018).

S46 Yang, S. *et al.* Highly-anisotropic optical and electrical properties in layered SnSe. *Nano Res.* **11**, 554-564 (2018).

S47 Guo, J. *et al.* Few-layer GeAs field-effect transistors and infrared photodetectors. *Adv. Mater.* **30**, 1705934 (2018).

S48 Kim, T. *et al.* High-mobility junction field-effect transistor via graphene/MoS2 heterointerface. *Sci. Rep.* **10**, 13101 (2020).

S49 Bao, Z. *et al.* Organic field-effect transistors with high mobility based on copper phthalocyanine. *Appl. Phys. Lett.* **69**, 3066-3068 (1996).

S50 Ong, B. S. *et al.* Stable, solution-processed, high-mobility ZnO thin-film transistors. *J. Am. Chem. Soc.* **129**, 2750-2751 (2007).

S51 Oh, G. *et al.* Graphene/pentacene barristor with ion-gel gate dielectric: flexible ambipolar transistor with high mobility and on/off ratio. *ACS Nano* **9**, 7515-7522 (2015).

S52 Kim, S. H. *et al.* Electrolyte-gated transistors for organic and printed electronics. *Adv. Mater.* **25**, 1822-1846 (2013).

S53 Li, J. *et al.* A stable solution-processed polymer semiconductor with record high-mobility for printed transistors. *Sci. Rep.* **2**, 754 (2012).

S54 Chiang, H. *et al.* High mobility transparent thin-film transistors with amorphous zinc tin oxide channel layer. *Appl. Phys. Lett.* **86**, 013503 (2005).

S55 Yabuta, H. *et al.* High-mobility thin-film transistor with amorphous InGaZnO4 channel fabricated by room temperature rf-magnetron sputtering. *Appl. Phys. Lett.* **89**, 112123 (2006).
